# CD8^+^ T cells provide immune protection against murine disseminated endotheliotropic *Orientia tsutsugamushi* infection

**DOI:** 10.1371/journal.pntd.0005763

**Published:** 2017-07-19

**Authors:** Guang Xu, Nicole L. Mendell, Yuejin Liang, Thomas R. Shelite, Yenny Goez-Rivillas, Lynn Soong, Donald H. Bouyer, David H. Walker

**Affiliations:** 1 Department of Pathology, The University of Texas Medical Branch, Galveston, Texas, United States of America; 2 Department of Microbiology and Immunology, The University of Texas Medical Branch, Galveston, Texas, United States of America; 3 Department of Internal Medicine, Division of Infectious Diseases, The University of Texas Medical Branch, Galveston, Texas, United States of America; Institut Pasteur, FRANCE

## Abstract

Scrub typhus, caused by a Gram-negative obligately intracellular coccobacillus, *Orientia tsutsugamushi*, is a long neglected but important tropical disease. *Orientia tsutsugamushi* causes illness in one million people each year, and 1 billion people are at risk. Without appropriate diagnosis and treatment, the disease can cause severe multiorgan failure with a case fatality rate of 7–15%. The current gaps in knowledge of immunity include the unknown mechanisms of host immunity to *O*. *tsutsugamushi*. Using an intravenous (i.v.) disseminated infection mouse model, we observed that more CD8^+^ T cells than CD4^+^ T cells were present in the spleen of infected mice at 12 dpi. We also determined that T_reg_ cells and the proportion of T cells producing IL-10 were significantly increased from 6 dpi, which correlated with the onset of illness, body weight loss, and increased bacterial loads. We further studied CD8^-/-^, MHC I^-/-^ and wild type control (WT) C57BL/6J mice to determine the importance of CD8^+^ T cells and MHC I molecules. After infection with an ordinarily sub-lethal dose of *O*. *tsutsugamushi*, all CD8^-/-^ and MHC I^-/-^ mice were moribund between 12 and 15 dpi, whereas all WT mice survived. Bacterial loads in the lung, kidney, liver and spleen of CD8^-/-^ and MHC I^-/-^ mice were significantly greater than those in WT mice. Interferon-γ (IFN-γ) and granzyme B mRNA levels in the liver of CD8^-/-^ and MHC I^-/-^ mice were significantly greater than in WT mice. In addition, more severe histopathologic lesions were observed in CD8^-/-^ mice. Finally, adoptive transfer confirmed a major role of immune CD8^+^ T cells as well as a less effective contribution by immune CD8 T cell-depleted splenocytes in protection against *O*. *tsutsugamushi* infection. These studies demonstrated the critical importance of CD8^+^ T cells in the host immune response during *O*. *tsutsugamushi* infection.

## Author summary

Scrub typhus is a febrile illness that can be life-threatening without appropriate treatment. It is caused by a gram-negative, obligately intracellular bacterium, *Orientia tsutsugamushi*. Approximately one million people annually are infected globally, mainly in the Asia-Pacific area. Unfortunately, no vaccine has been developed for the prevention of the disease. There is very little known about the pathogenesis of or host immunity to scrub typhus. The limited number of studies on the mechanisms of immunity has failed to provide a fundamental explanation of the protective host immune response during the disease due in part to the outdated and inaccurate animal models employed. Here, we examined and confirmed that CD8^+^ T cells provide critical protection during the infection. Our data also demonstrated the potential contribution of non-CD8^+^ immune cells in protection against the infection. This study of host immune responses during scrub typhus with a more accurate mouse model improves the understanding of the interaction between the host and pathogen, and provides a foundation for future investigations of pathogenesis, immune control and prevention including vaccine development.

## Introduction

Scrub typhus is a serious public health problem in Asia and the islands of the western Pacific and Indian oceans, causing illness in one million persons each year [1]. Recent studies have identified scrub typhus in South America and Africa [2,3,4]. The causative agent of scrub typhus is *Orientia tsutsugamushi*, a gram-negative obligately intracellular bacillus [5,6]. The acute undifferentiated febrile illness caused by scrub typhus poses a significant threat to public health in the endemic area [7]. However, the study of *O*. *tsutsugamushi* has been largely neglected for decades. Though *Orientia* belongs to the Rickettsiaceae family, there are major differences between the two genera, *Rickettsia* and *Orientia*. For instance, *O*. *tsutsugamushi* does not contain lipopolysaccharide, or have a surrounding electron-lucent zone [1,8]. It has a thicker outer cell wall leaflet and distinctive outer membrane proteins. *Orientia* spreads more slowly and buds from the host cytoplasmic membrane [9]. In addition, it has strain-specific antigens, which make cross protection much less effective than in infections caused by *Rickettsia* [10,11].

Because of the overlapping clinical manifestations with other common febrile illnesses as well as limitations of current diagnostic methods, the clinical diagnosis of scrub typhus is difficult. The absence of an early diagnosis impedes timely appropriate therapy. There is still a significant gap in understanding of how the pathogen invades, disseminates, and interacts within the host [12]. The recent reemergence of scrub typhus and appearance of antimicrobial resistance emphasize the need for the development of effective preventive measures including a vaccine, which requires understanding the mechanisms of the host immune response to the invasion, infection and persistence of *O*. *tsutsugamushi*. *Orientia tsutsugamushi* can persist chronically in human patients even when treated with antibiotics [13,14], but the mechanisms underlying the early and late interactions between the pathogen and the host are still unclear. Both innate and adaptive immunity are involved in control of *Orientia* infection, but more systematic studies with appropriate animal models and clinical studies are still lacking [6,15,16]. For instance, Hauptmann et al. recently found the importance of CD8^+^ T cells during *Orientia* infection with their combined subcutaneous (s.c.) and intradermal (i.d.) footpad inoculation mouse model [17]. The study provided a foundation for future work; however, there are elements of the animal models and experimental design that require further investigations.

The widely used intraperitoneal (i.p.) inoculation route produces an infection of the peritoneal cavity that results in severe mesothelial cell infection and fatal *Orientia* peritonitis instead of disseminated, systemic endothelial cell-targeted infections observed in scrub typhus in human patients [18,19,20,21,22,23]. Recently, our laboratory has developed new mouse models for *Orientia* infection, which better mimic the endothelial and macrophage target cells, organ distribution, disease course, pathology and immune responses of human patients [24,25]. We observed human scrub typhus-like disease development, such as disseminated endothelial infection and injury, lymphohistiocytic vasculitis, interstitial pneumonitis and hepatic damage, with our intravenous (i.v.) and intradermal (i.d.) inoculation models [24,25,26].

As a long neglected and understudied pathogen, there are still many unknowns and contradictions regarding the pathogenesis of *O*. *tsutsugamushi* infection and the host immune responses to the infection. During *Orientia* infection of humans, the bacteria infect endothelial cells, macrophages and dendritic cells *in vivo*, and their infection of other cell types has not been well verified [22,27,28,29]. Paris et al. discovered that dendritic cells are the primary targets in the inoculation site eschar during feeding by an *O*. *tsutsugamushi*-infected mite [12], but during dissemination endothelial cells are the main target of *Orientia* infection [22,30,31]. There have been two publications claiming the infection of hepatocytes, but the evidence is not convincing [32,33]. The structures in Pongponratn et al have cell wall ultrastructure that is completely different from *Orientia*. The staining in Chung et al. includes non-specific staining and lacks the morphology of *Orientia*. Furthermore, it is not ideal or accurate to draw conclusions about host immune responses during *O*. *tsutsugamushi* infection from *in vitro* studies using non-target cells, such as epithelial cells, instead of the main target cells documented in systemic infection.

As an obligately intracellular pathogen, it is reasonable to propose that cellular immunity including CD8^+^ T cells plays critical roles in host defense and clearance of *O*. *tsutsugamushi* infection [15,34,35,36]. The *in vitro* study by Rollwagen et al. showed that immune splenocytes lysed *Orientia-*infected L929 fibroblasts [37]. In a clinical study, granzyme, interferon-γ (IFN-γ) inducible protein 10, and monokine induced by IFN-γ were elevated in human scrub typhus patients [38]. In addition, previous studies of *Rickettsia* infection in our laboratory demonstrated that T lymphocytes not only secrete IFN-γ, but also provide important immune cytotoxic activity effectors to eliminate rickettsiae [39]. Our laboratory confirmed the effectiveness of both CD4^+^ and CD8^+^ T lymphocytes, especially CD8^+^ T cells, in *Rickettsia* infection and clearance through depletion and adoptive transfer of immune CD4^+^ or CD8^+^ T lymphocytes [40]. This led to our hypothesis that T lymphocytes, especially CD8^+^ T cells, are critical to protect the host against *Orientia* infection.

In order to better understand the host immune responses during *Orientia* infection, we analyzed the changes in host immune responses during the development of *O*. *tsutsugamushi* infection in our newly developed i.v. inoculation mouse model. We examined the roles of CD8^+^ T lymphocytes, non-CD8 spleen cells, and major histocompatibility complex (MHC) class I in *Orientia* infection. Our adoptive transfer of immune CD8^+^ T cells or non-CD8 splenocytes demonstrated the critical role of CD8^+^ T cells, as well as the contribution of other immune cells, in protection against the infection. Our research provides a foundation for better understanding of host immunity against and pathogenesis of *O*. *tsutsugamushi* infection. Knowledge of these mechanisms will facilitate development of innovative therapeutic approaches including a vaccine for prevention of scrub typhus.

## Materials and methods

### Ethics statement

All procedures and experiments were reviewed and approved by the Institutional Animal Care and Use Committee (IACUC) of the University of Texas Medical Branch (UTMB) (Protocols: 9007082, and 1302003) in accordance with Guidelines for Biosafety in Microbiological and Biomedical Laboratories. UTMB operates to comply with the USDA Animal Welfare Act (Public Law 89–544), the Health Research Extension Act of 1985 (Public Law 99–158), the Public Health Service Policy on Humane Care and Use of Laboratory Animals, and the NAS Guide for the Care and Use of Laboratory Animals (ISBN-13). UTMB is a registered research facility under the Animal Welfare Act, and has a current assurance on file with the Office of Laboratory Animal Welfare, in compliance with NIH Policy.

### Animals

C57BL/6J (B6), B6.129S2-Cd8a^tm1Mak^/J (CD8^-/-^), and B6.129P2-B2m^tm1Unc^/J (MHC I^-/-^) mice were purchased from the Jackson Laboratory. Age- and gender-matched, 7–12 week old mice were used in all studies. Experimental mice were housed in the animal biosafety level 3 facility at UTMB. Mice were inoculated i.v. with ~1.25×10^6^ focus forming units (FFU) of *Orientia* as a sub-lethal dose, or ~1.64×10^7^ FFU as a lethal dose. All mice were monitored three times each day after infection, and euthanized at selected time points. Moribund mice euthanized for animal welfare were counted as deceased for statistical analyses.

### Bacterial culture

As described previously [24], we cultivated *O*. *tsutsugamushi* Karp strain in Vero cells or C57BL/6J mice (Jackson Laboratory, ME, USA). Briefly, the bacteria were added to T150 cell culture flasks containing confluent monolayers of Vero cells. The cells were cultivated in Dulbecco’s Modified Eagle’s Medium (DMEM, Gibco, MA, USA) with 3% fetal bovine serum (FBS, HyClone, PA, USA) and 1% HEPES buffer (Gibco, MA, USA). Smears of infected cells stained with Diff-Quik (Fisher Scientific, MA, USA) were prepared to assess the level of infection when rounded and floating cells were observed between 14–21 days post-infection. The infected cells were harvested by high speed centrifugation at 22,000× *g* for 45 minutes at 4°C when 80–90% of Vero cells were infected. Sucrose phosphate glutamate buffer (SPG, 218 mM sucrose, 3.8 mM KH_2_PO_4_, 7.1 mM K_2_HPO_4_, 4.9 mM monosodium L-glutamic acid) was used to resuspend the pellet. Glass beads were used to release the intracellular bacteria from infected cells, and the supernatant was collected after centrifugation at 700× *g* for 5 minutes. Another high speed centrifugation concentrated the stock, which was stored at -80°C in SPG for subsequent use.

The animal passages were performed as previously described [24]. Naive C57BL/6J mice were inoculated i.v. with *O*. *tsutsugamushi* and were euthanized when they exhibited signs of illness. The livers were aseptically collected and homogenized with cold SPG buffer. Supernatant was collected after two 5-minute centrifugations at 700× *g*. A 45-minute high speed centrifugation at 22,000× *g* and 4°C was employed to concentrate the stock. All stocks were stored at −80°C in SPG in the biosafety level-3 facility.

### Bacterial load determination

Bacterial loads were determined by quantitative real-time PCR [41]. Qiagen DNeasy Kits (Qiagen, Hilden, Germany) were used to extract DNA from the lung, liver, kidney, spleen, and other tissue samples. We used the *O*. *tsutsugamushi* 47-kDa gene to identify the bacteria and determine the bacterial loads [41]. The primers were OtsuF630 (5’-AACTGATTTTATTCAAACTAATGCTGCT-3’) and OtsuR747 (5’-TATGCCTGAGTAAGATACGTGAATGGAATT-3’) (Integrated DNA Technologies, IA, USA). The conditions of qPCR were 3 minutes at 94°C, and 40 cycles of 95°C for 5 seconds, 60°C for 30 seconds. We used total nanogram (ng) of DNA per μL to normalize the concentration of the bacterial loads [24,41].

### Flow cytometry

The spleens of *Orientia*-infected mice or uninfected control mice were collected and homogenized with phosphate buffered saline. Red Blood Cell Lysis Buffer (Sigma-Aldrich, MO, USA) was used for the lysis of red blood cells. Samples were exposed to cell stimulation cocktail (Life Technologies, CA, USA) and GolgiPlug protein transport inhibitor (BD Bioscience, CA, USA) at 37°C for 4 hours. The samples were first stained using Live/Dead cell stain and surface antibodies, followed by intracellular staining with the Foxp3 Transcription Factor Staining Buffer Set (Life Technologies, CA, USA). The anti-mouse antibodies and live cell dye were from either Life Technologies or BD Bioscience: LIVE/DEAD Fixable Near-IR Dead Cell Stain Kit, purified anti-CD16/CD32, eFluor 450-labeled anti-CD3, PE-Cy7-labeled anti-CD25, Alexa Fluor (AF) 700-labeled anti-CD4, AF 488-labeled anti-CD8a, PE-CF594-labeled anti-FoxP3, and PE-labeled anti-IL-10. Data were collected by a BD LSRII Fortessa cell analyzer (BD Bioscience, CA, USA) and analyzed with FlowJo 10 (FlowJo LLC, OR, USA).

### Quantitative reverse transcriptase PCR (qRT-PCR) analysis

Quantitative reverse transcriptase PCR (qRT-PCR) was used to analyze the mRNA levels of cytokines and chemokines (S1 Table) [15]. The tissues were collected and placed in RNALater (Ambion, MA, USA) at 4°C for 24 hours before being stored at -80°C. RNeasy minikits and RNase-free DNase Set (Qiagen, Hilden, Germany) were used to extract and purify total RNA. iScript cDNA synthesis kit (Bio-Rad Laboratories, CA, USA) was used to synthesize cDNA. The abundance of target genes was determined by qRT-PCR with Bio-Rad CFX 96 Real-Time System and SYBR Green Supermix (Bio-Rad Laboratories, CA, USA). The PCR incubations were 3 minutes at 95°C, and 40 cycles of 95°C for 10 seconds, 60°C for 10 seconds, followed by 10 seconds at 72°C. Dissociation melting curves were obtained to confirm the purity of final products. The 2^-ΔΔCt^ method was used to calculate the relative abundance of mRNA levels with glyceraldehyde-3-phosphate dehydrogenase (GAPDH, (F, 5’-CAACTACATGGTCTACATGTTC-3’; R, 5’-CTCGCTCCTGGAAGATG-3’, IDT) as housekeeping gene denominator [15,42]. All qRT-PCR reactions were duplicated.

### Serum biochemistry

Whole blood was collected in MiniCollect Z serum tubes (Greiner bio-one, Frickenhausen, Germany) and serum was separated by centrifugation 3000x*g* for 10 minutes to determine the blood chemistry profile. Albumin, alanine aminotransferase and aspartate aminotransferase were measured by the clinical core laboratory at the Department of Pathology, UTMB, Texas, USA.

### Histopathology and immunohistochemistry

The tissues were fixed in 10% neutral buffered formalin and embedded in paraffin. Hematoxylin and eosin were used to stain the tissue sections (5 μm thickness). All slides were reviewed and scored by four scientists trained in histopathology. We used a 0 to 4 scoring system (0, normal; 1, cellular infiltration presence; 2, multi-focal lesions; 3, no more than 2 areas of necrosis; 4, more than 2 foci of necrosis in one field) to assess the histopathology in the liver. Tissue sections from the experimental animals were stained with ApopTag Peroxidase *In Situ* Apoptosis Detection Kit (Millipore, Darmstadt, Germany) following the manufacturer’s protocol with hematoxylin used instead of methyl green in the counterstaining step. The scoring system for apoptosis staining was a 0 to 5 point scale (0, no apoptosis; 1, no more than 10 apoptotic staining positive cells per 100x field; 2, between 11 and 20 apoptotic cells per 100x field; 3, between 21 and 30 apoptotic cells per 100x field; 4, between 31 and 40 apoptotic cells per 100x field; 5, more than 40 apoptotic cells per 100x field).

### Adoptive transfer of splenocytes

In this experiment, a sublethal dose (1.25×10^6^ FFU) of *O*. *tsutsugamushi* Karp strain and the same dose given as a booster were inoculated into C57BL/6J mice 27 days and 7 days, respectively, before the spleen of the donor mouse was harvested [39,40]. The spleens of uninfected mice of the same age were used to prepare nonimmune control cells. CD8^+^ T lymphocytes were then isolated by the negative magnetic CD8^+^ T cell isolation kit (Miltenyi Biotec, Bergisch Gladbach, Germany). We also collected the CD8 T cell-depleted splenocytes from the column following the manufacturer’s instructions. The splenocytes were pooled and counted before adoptive transfer. One spleen-equivalent of separated CD8^+^ T cells or CD8 T cell-depleted splenocytes (~10^7^ cells) was transferred to recipient B6 mice via the i.v. route. After 24 hours, all recipient mice were challenged i.v. with an ordinarily lethal dose (10 LD_50_: 8.25×10^7^ FFU) of *O*. *tsutsugamushi*. The negative magnetic selection generated more than 91% purity of CD8^+^ T cells with less than 0.5% CD4^+^ T cells. There were 20%-27% CD4^+^ T cells and less than 2% CD8^+^ T cells among the CD8 T cell-depleted splenocytes.

### Statistical analysis

Results are presented either as mean ± SD or as median and range, as indicated. Analyses were performed with independent samples T test and one-way ANOVA with Tukey’s multiple comparison as post-hoc analysis. P < 0.05 was considered to be statistically significant. GraphPad Prism 5.0 and SPSS 18.0 were used for statistical calculations.

## Results

### *Orientia tsutsugamushi* infection resulted in proliferation of CD8^+^ T cells, T_reg_ cells, and IL-10 producing T cells

**Fig 1:**
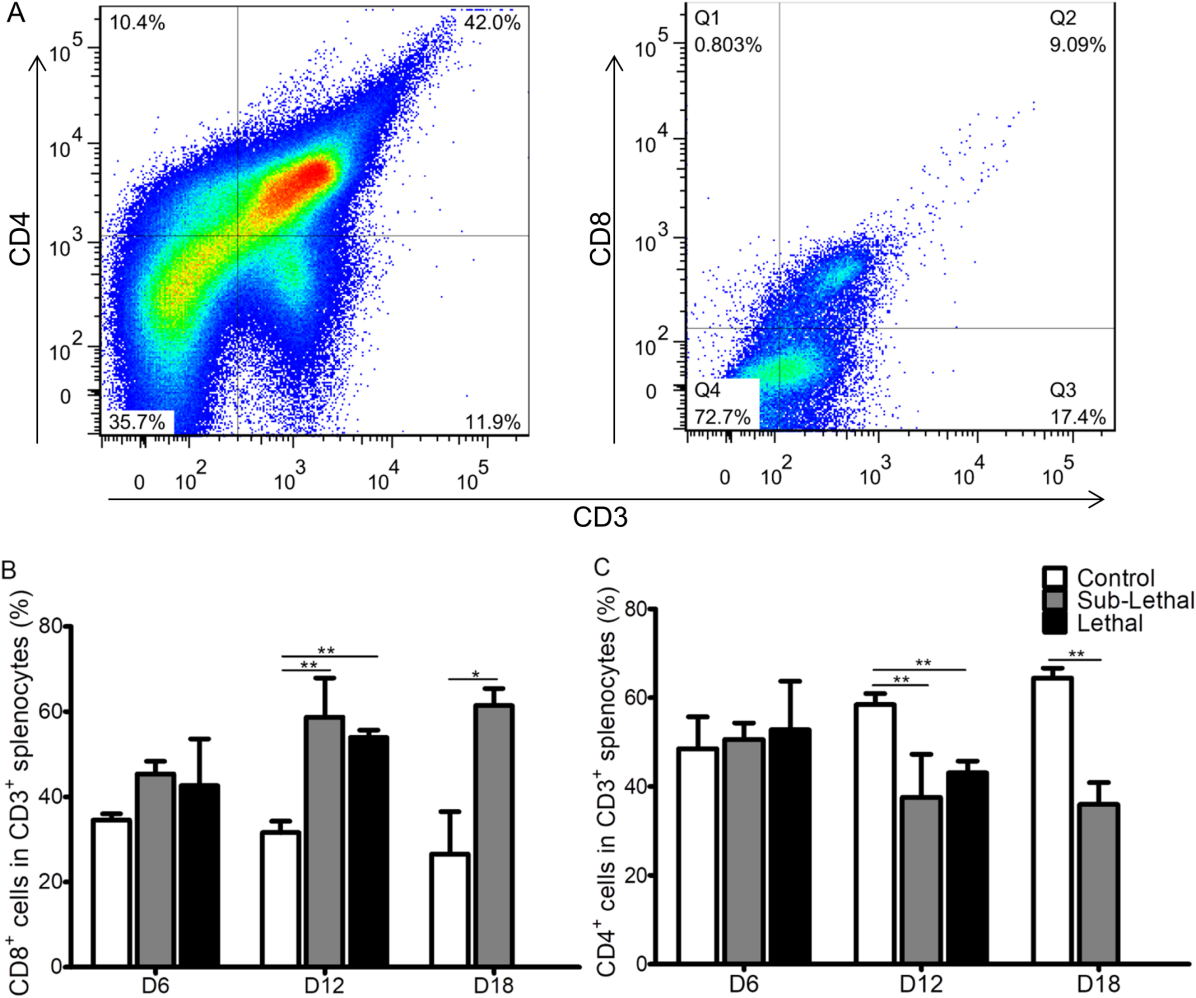
T cell subsets in *Orientia-*infected mice. Using the gating strategy (**A**), we observed that more CD8^+^ T cells (**B**) than CD4^+^ T cells (**C**) were induced in wild type C57BL/6J (WT) mice at 12 days post sublethal or lethal *Orientia* infection. PBS was used as control inoculum. Data are expressed as mean ± SD. *, p<0.05; **, p<0.01.

To determine the host immune responses during *Orientia* infection, we inoculated three groups of C57BL/6J mice i.v. with PBS, a sublethal dose, or a lethal dose of *O*. *tsutsugamushi* Karp strain. All mice challenged with a lethal dose were moribund by 12 days post-infection (dpi). On 6, 12 and 18 dpi, the spleens were collected and processed for flow cytometric analysis as described above. The percentages of neither CD4^+^ T cells nor CD8^+^ T cells from both sublethal and lethal dose-challenged mouse spleens were significantly greater than PBS inoculated control mice on 6 dpi (**Fig 1**). However, we observed that both infected groups had considerably increased spleen size after 12 dpi. Our flow cytometry data further demonstrated that more CD8^+^ T cells than CD4^+^ T cells were induced in infected mice at 12 and 18 dpi (**Fig 1**). We also determined that the proportions of T_reg_ cells and T cells producing IL-10 were increased from 6 dpi, but the proportions of both of them were reduced after 12 dpi (**S1 Fig**). Our data showed that the adaptive immune response after *Orientia* infection was skewed toward CD8^+^ T cell proliferation, and regulatory immune responses were induced at an early stage. These results suggested that CD8^+^ T cells may play important roles in host immunity against *Orientia* infection.

### CD8^-/-^ mice lost more weight and were more susceptible to *O*. *tsutsugamushi* than WT mice

**Fig 2:**
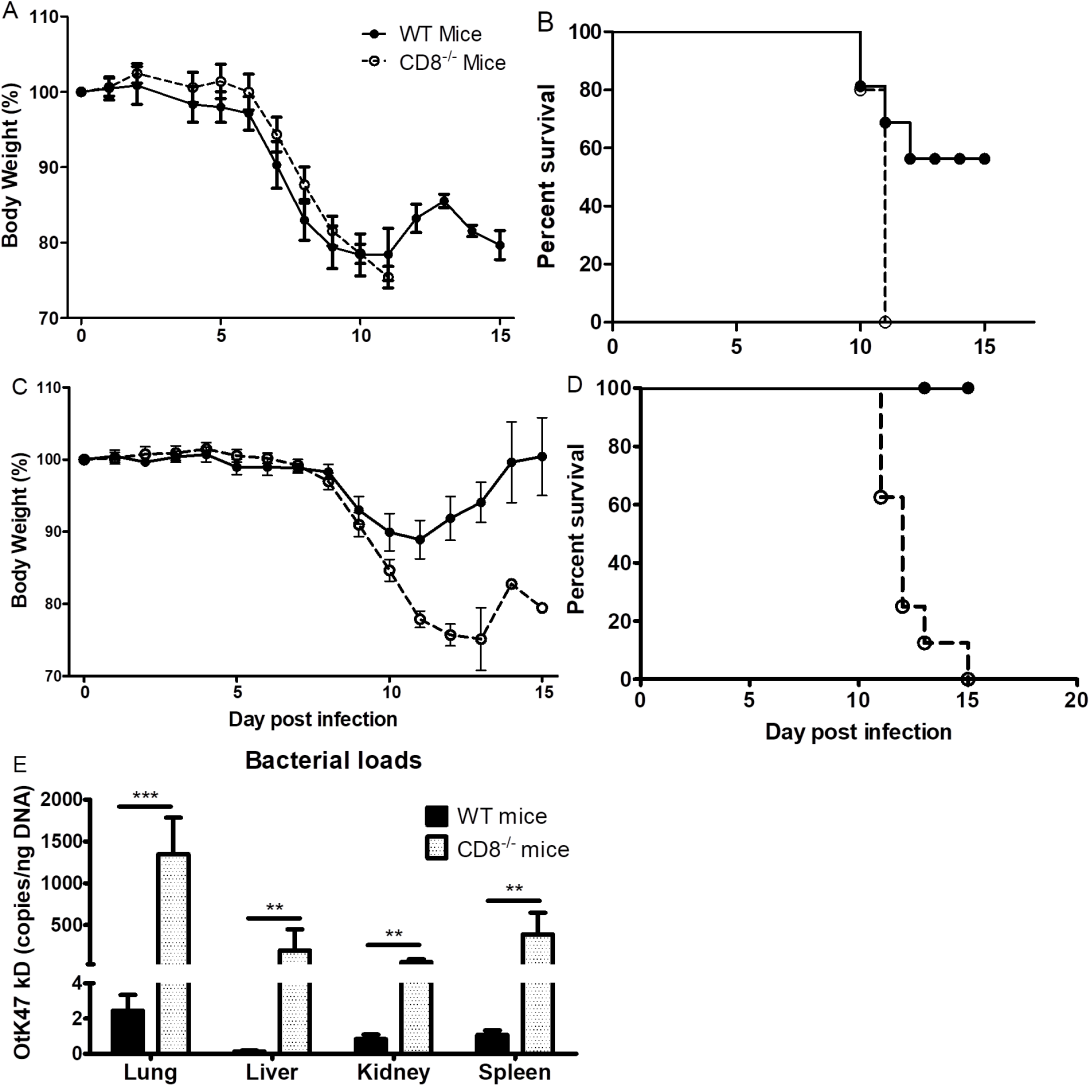
Body weight change, survival, and bacterial loads of CD8^-/-^ mice and WT mice infected with *O*. *tsutsugamushi*. Mice challenged with 1 LD_50_ of *O*. *tsutsugamushi* began losing weight 7 dpi concurrent with signs of illness (**A**). Half of the WT mice (solid circles) were moribund between days 10 and 12 while all CD8^-/-^ mice (open circles) were moribund by 11 dpi (**B**). Weight change (**C**) and survival curve (**D**) of mice following challenge with a sublethal dose of *Orientia*. None of the WT mice expired while all CD8^-/-^ mice were moribund between 11 and 15 dpi (**D**). QPCR demonstrated that CD8^-/-^ mice infected with a sublethal dose of *O*. *tsutsugamushi* had significantly higher bacterial loads in the lung, liver, kidney, and spleen than WT mice at 12 dpi (**E**). *, p<0.05; **, p<0.01; ***, p<0.001, n = 8.

We first infected both CD8^-/-^ mice and age-matched WT mice i.v. with one LD_50_ (8.25×10^6^ FFU) of *O*. *tsutsugamushi* Karp strain. Both groups of mice maintained stable weight until 6 dpi (**Fig 2A**). We observed signs of illness including hunched back, ruffled fur, erythema, and eye secretions that coincided with weight loss beginning at 7 dpi. CD8^-/-^ mice lost more weight than their WT counterparts from 9 dpi (**Fig 2**). All CD8^-/-^ mice were moribund by day 11 post-infection while, as expected, only half of the WT mice succumbed to infection (**Fig 2B**). The WT survivors began to gain weight after 11 dpi, and maintained their weight after 14 dpi (**Fig 2A**).

To better understand the effects of CD8^+^ T cells during *O*. *tsutsugamushi* infection, we repeated the experiment with a sublethal i.v. dose (1.25×10^6^ FFU) challenge of *O*. *tsutsugamushi* Karp strain. Similar to the one LD_50_ dose-challenged mice, both sublethal dose challenged WT and CD8^-/-^ mice showed similar weight change and other signs of sickness during the early stage. Both groups of infected mice in this study began losing weight at 9 dpi, and CD8^-/-^ mice continued losing weight until they became moribund (11–15 dpi) while WT mice ceased losing weight on 11 dpi and recovered (**Fig 2C and 2D**).

We further used quantitative real-time PCR to determine the bacterial loads in different organs of the sublethally infected CD8^-/-^ mice and WT mice. There were significantly higher bacterial loads in the lung, kidney, liver, and spleen of CD8^-/-^ mice than in WT mice on day 12 of infection (**Fig 2E**). These studies indicated that CD8^-/-^ mice were more susceptible to *Orientia* infection than WT mice.

### Deficiency of CD8^+^ T cells resulted in an increase of IFN-γ and granzyme B mRNA levels in the liver of infected mice

**Fig 3:**
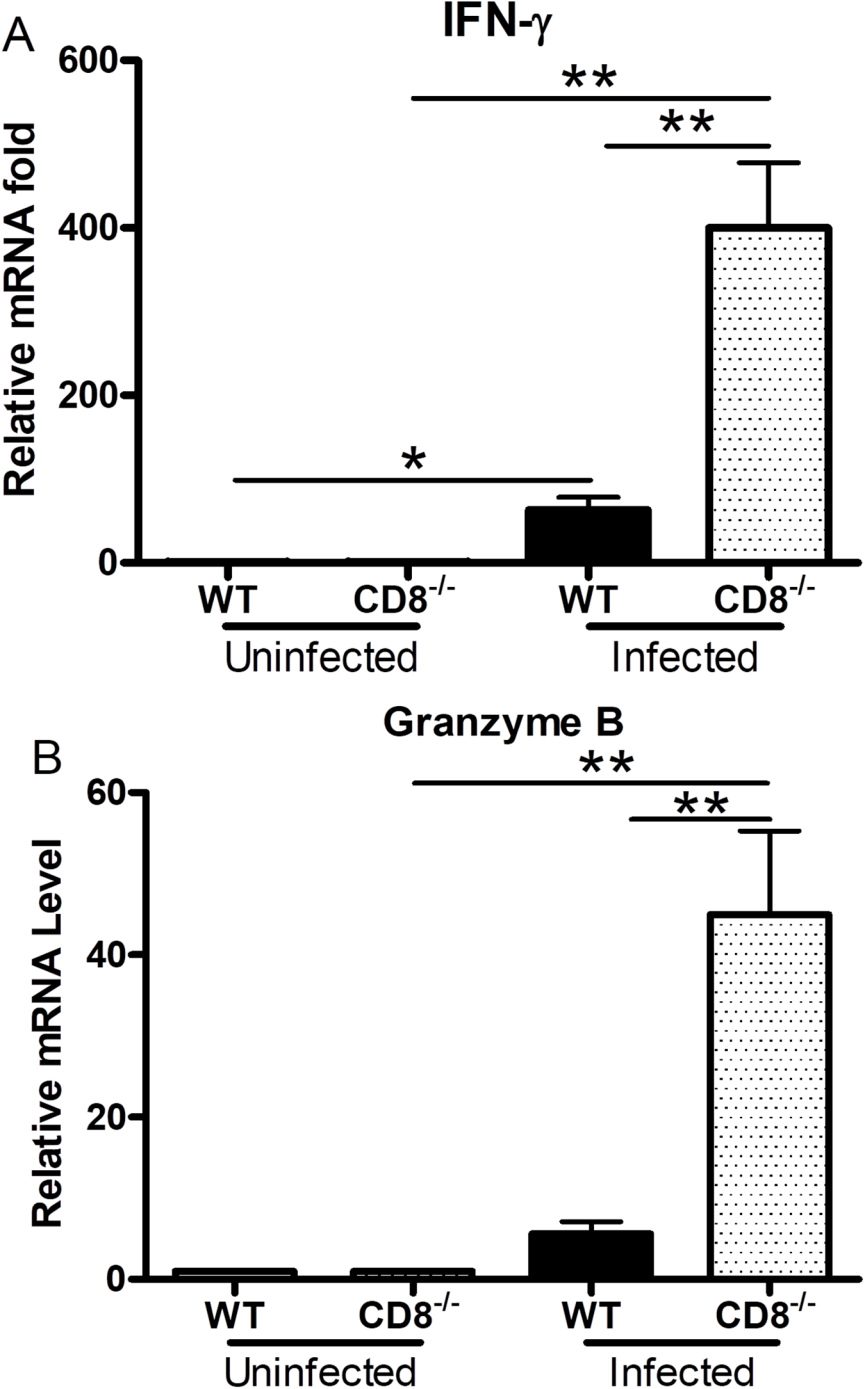
Gene expression in the liver of CD8^-/-^ and WT mice infected with *O*. *tsutsugamushi*. There was significantly greater IFN-γ (**A**) and granzyme B (**B**) mRNA in the liver of infected CD8^-/-^ mice than in the WT mice at 12 dpi. Data are shown as mean ± SD in each group and are presented as relative mRNA levels with the 2^-Δ ΔCt^ of housekeeping genes normalization method. *, p<0.05; **, p<0.01, n = 8; all PCRs were duplicated.

To further understand the host immune responses during *Orientia* infection, we employed qRT-PCR to determine the mRNA levels of cytokines among different organs in infected CD8^-/-^ mice and WT mice. The mRNA levels of the important pro-inflammatory cytokine IFN-γ was significantly higher in the liver of infected CD8^-/-^ mice than in the infected WT control at 12 dpi (p value = 0.0037, **Fig 3A**). Significantly higher mRNA levels of IFN-γ were observed in both infected CD8^-/-^ and WT mice than in corresponding uninfected mice (WT: p = 0.0137; CD8^-/-^: p = 0.0013, **Fig 3A**). There were no significant differences in the IFN-γ mRNA levels in the spleen, kidney and liver of the uninfected knockout (KO) and WT mice. Both groups of infected mice had higher levels of IFN-γ mRNA in the lung than those in uninfected mice. We measured granzyme B because it is secreted by CD8^+^ T lymphocytes and natural killer (NK) cells to mediate apoptosis of cells infected with intracellular pathogens and tumor cells [43]. Our qRT-PCR studies demonstrated that the granzyme B mRNA levels in the liver of infected CD8^-/-^ mice were significantly higher than in the infected WT mice at 12 dpi (p = 0.0092, **Fig 3B**). Infected CD8^-/-^ mice had significantly greater mRNA levels of granzyme B than uninfected CD8^-/-^ mice (p = 0.0048, **Fig 3B**). CXCL-10 is anIFN-γ-inducible monokine that can recruit primed T lymphocytes and NK cells to the site of inflammation [44,45]. We observed that CD8^-/-^ mice had significantly lower level of CXCL-10 (**S2 Fig**). No statistically significant differences were detected in the levels of TNF-α, IL-10, MCP-1, and MIP-2 between CD8^-/-^ and WT mice (**S2 and S3 Figs**).

### CD8^-/-^ mice infected with *O*. *tsutsugamushi* had more severe liver damage

**Fig 4:**
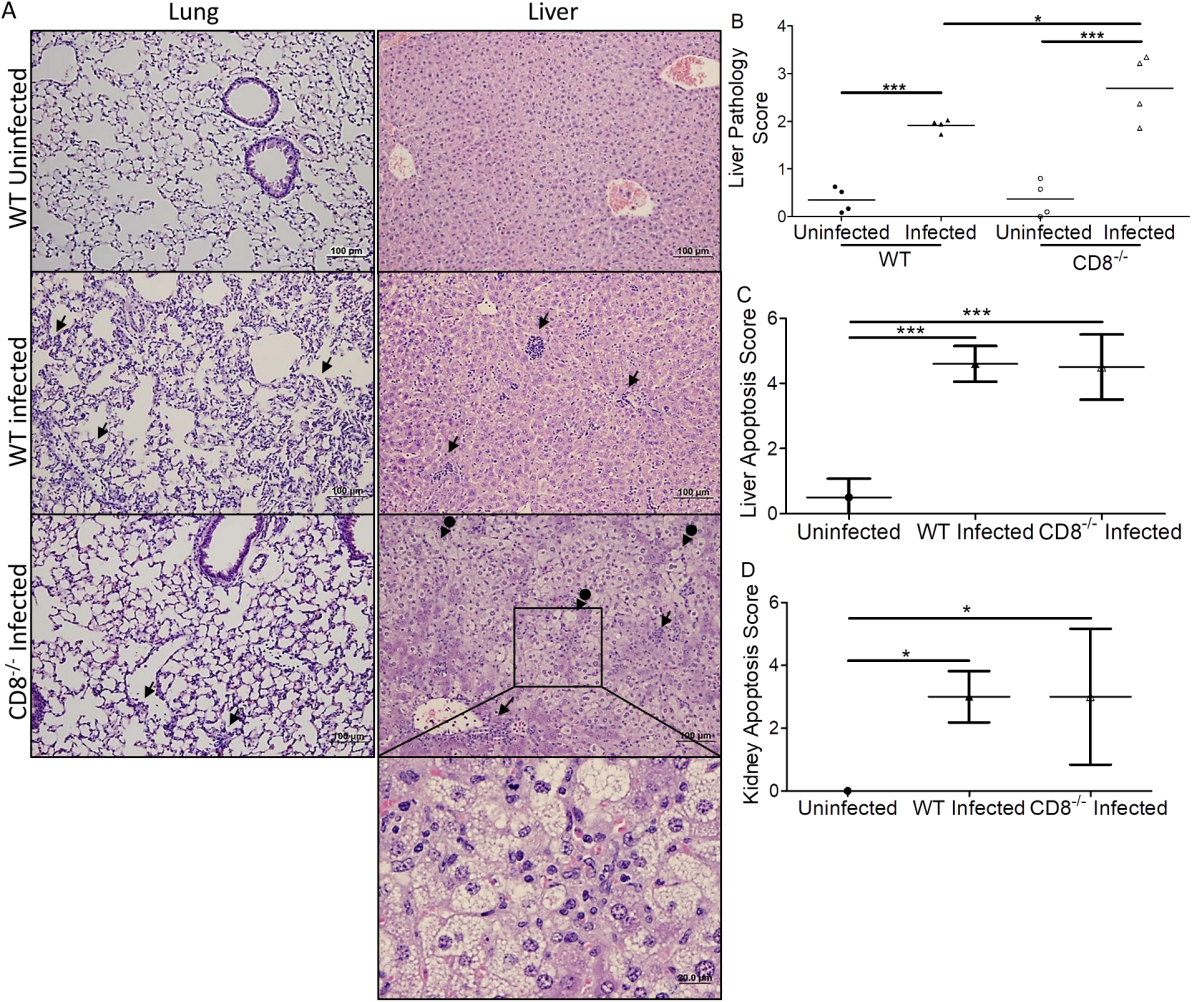
Histopathological comparison of CD8^-/-^ and WT mice infected with *O*. *tsutsugamushi*. More cellular infiltrations (arrows) were found in the lung and liver (**A**, mag: 100x, 400x inset) of WT mice than CD8^-/-^ mice. Increased necrosis and steatosis were observed in the liver of CD8^-/-^ mice (arrows with circle end). Higher liver pathology scores indicating injury were observed in infected WT and CD8^-/-^mice (**B**). The pathology score of infected CD8^-/-^ mice was greater than that of infected WT mice (**B**). There was no apoptosis score difference in the liver (**C**) or kidney (**D**) between infected WT and CD8^-/-^ mice, but significantly greater apoptosis was measured in all mice after *Orientia* infection. *, p<0.05; ***, p<0.001, n = 8; Each tissue sample was blindly scored by four experienced investigators.

Although we observed more cellular infiltrations in the lung, liver, kidney (**Fig 4A**), and heart of WT mice than in matched CD8^-/-^ mice, the infected CD8^-/-^ mice had a greater liver histopathology score than WT control mice (Pathology score 2.70 vs 1.92, p<0.05, **Fig 4B**). We further examined the levels of alanine transaminase (ALT), aspartate transaminase (AST), and albumin (ALB) in serum of naïve and infected WT, CD8^-/-^, and MHC I^-/-^ mice (**S4 Fig**). We observed significantly increased ALT and AST levels as well as decreased ALB in the serum of infected mice compared to uninfected control (**S4 Fig**). CD8^-/-^ mice trended towards increased ALT and AST. The livers of infected CD8^-/-^ mice also showed steatosis. We further used an Apoptosis Detection Kit to determine the difference in apoptosis during *Orientia* infection between CD8^-/-^ mice and WT mice. The immunohistochemical method clearly demonstrated that there were more apoptotic cells in the liver and kidney of all infected mice than in the uninfected control mice (**Fig 4C and 4D, S5 Fig.**). However, the apoptosis scores in liver and kidney of infected WT and CD8^-/-^ mice were not statistically different. In addition, mRNA levels of *Bcl*-2, which encodes an anti-apoptotic molecule, were similar in the liver between infected CD8^-/-^ mice and WT mice (**S5 Fig**).

### MHC I^-/-^ mice had increased severity of illness and greater susceptibility to *O*. *tsutsugamushi* infection than WT mice

**Fig 5:**
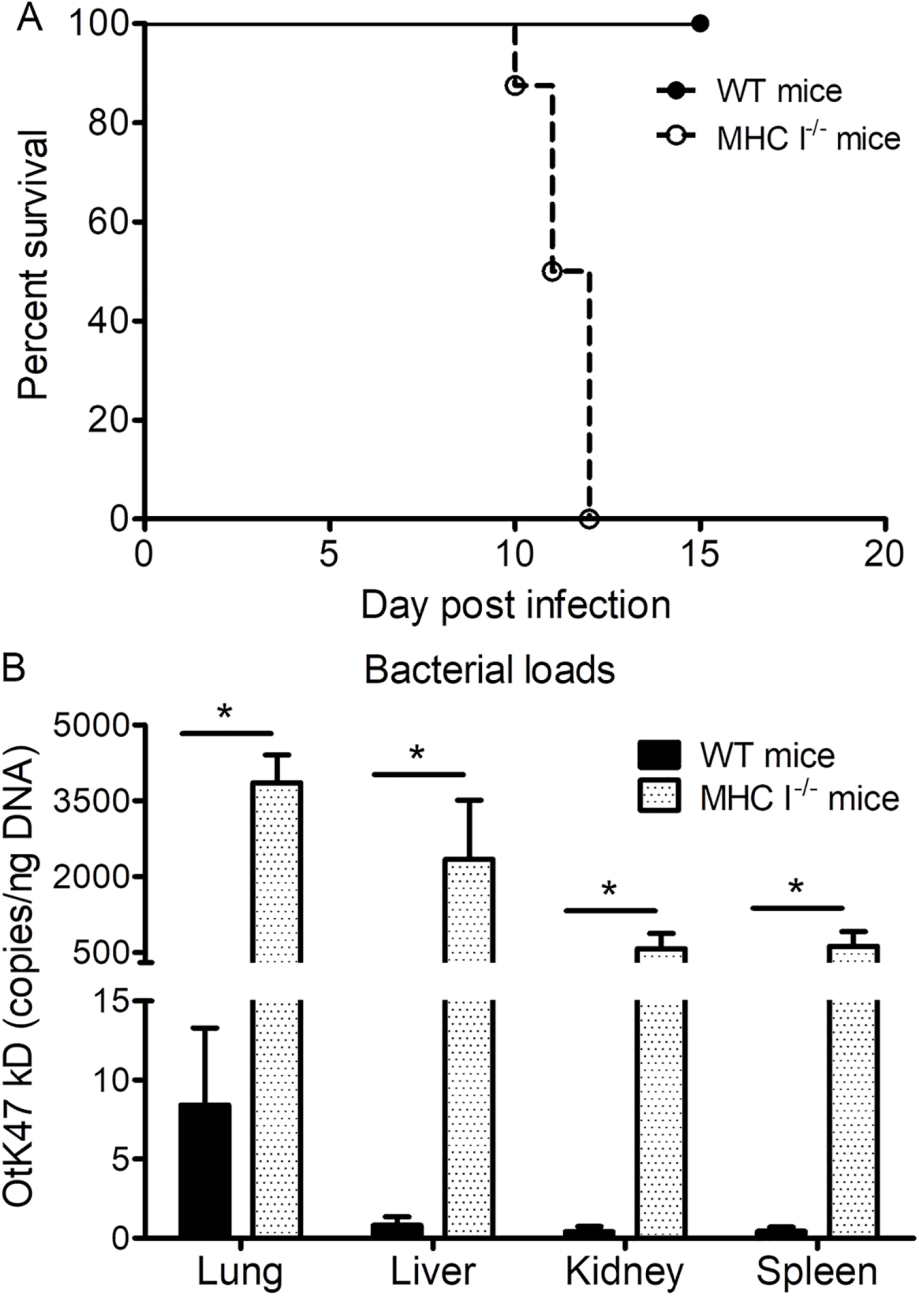
Survival and bacterial loads of MHC I^-/-^ mice and WT mice infected with *O*. *tsutsugamushi*. All infected MHC I^-/-^ mice (open circles) became moribund between 10 and 12 dpi while none of the infected WT mice (solid circles) expired (**A**). MHC I^-/-^ mice had significantly greater bacterial loads in the lung, liver, kidney, and spleen than in the WT mice at 11 dpi (**B**). *, p<0.05; n = 8.

To further investigate the role of CD8^+^ T cells and the upstream pathway, we used an ordinarily sublethal dose (1.25×10^6^ FFU) of *O*. *tsutsugamushi* Karp strain to infect MHC I^-/-^ mice and WT C57BL/6J mice via i.v. inoculation. Similar to the results of CD8^-/-^ mouse studies, both groups of mice maintained a stable weight until 8 dpi (**S6 Fig**). We observed signs of illness including hunched posture, ruffled fur, erythema, and ocular secretion coincident with the onset of weight loss on day 9. MHC I^-/-^ mice became more severely ill than WT C57BL/6J mice after 9 dpi. One MHC I^-/-^ mouse expired on 10 dpi, and the rest became moribund on 11 and 12 dpi. None of the C57BL/6J control mice became moribund, and they gained weight after 10 dpi (**Fig 5A and S6 Fig**).

Quantitative real-time PCR determination of bacterial loads in organs of MHC I^-/-^ mice and WT mice revealed significantly greater bacterial loads in the lung, kidney, liver, and spleen of MHC I^-/-^ mice than in the WT mice at 11 dpi (**Fig 5B**). We concluded that MHC I^-/-^ mice were more susceptible to *Orientia* infection.

### MHC I^-/-^ mice infected with *O*. *tsutsugamushi* had greater mRNA levels of IFN-γ and granzyme B in the liver

**Fig 6:**
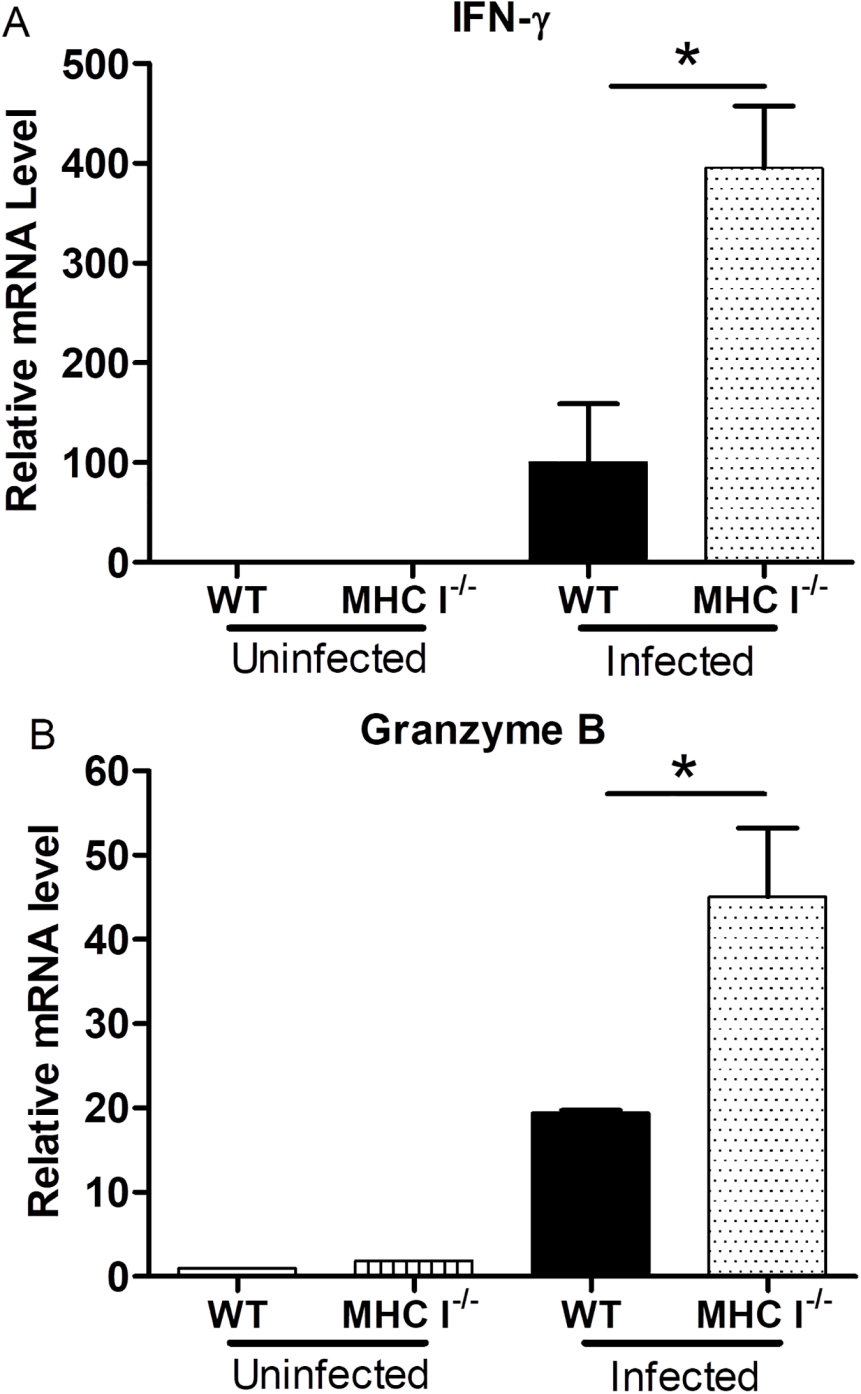
Gene expression in the liver of MHC I^-/-^ mice and WT mice infected with *O*. *tsutsugamushi*. There were significantly higher levels of IFN-γ (**A**) and granzyme B (**B**) mRNA in the liver of infected MHC I^-/-^ mice than in the WT mice at 11 dpi. Data are shown as mean ± SD in each group and presented as relative mRNA levels with the 2^-Δ ΔCt^ of housekeeping genes normalization method. *, p<0.05; **, p<0.01; all PCRs were duplicated.

We used qRT-PCR to determine the levels of IFN-γ, granzyme B, and CXCL-10 mRNA in different organs of infected MHC I^-/-^ mice and WT mice. Similar to CD8^-/-^ mice, infected MHC I^-/-^ mice had a significantly higher level of IFN-γ mRNA in the liver than infected WT mice at 11 dpi (p = 0.0204, **Fig 6A**). We also observed that infected MHC I^-/-^ mice had significantly higher mRNA levels of IFN-γ than in uninfected MHC I^-/-^ mice (p = 0.0078, **Fig 6B**). The granzyme B mRNA levels in the infected MHC I^-/-^ mice, similar to the observations in CD8^-/-^ mice, were higher than those in infected WT mice (p = 0.0447, **Fig 6B**). Infected MHC I^-/-^ mice also had significantly greater mRNA levels of granzyme B than their uninfected controls (p = 0.0116, **Fig 6B**). CXCL-10 mRNA was significantly higher in the liver of infected MHC I^-/-^ mice than in the WT mice at 11 dpi (p = 0.0041, **S7 Fig**). No significant differences were observed between uninfected MHC I^-/-^ and uninfected WT mice in the levels of IFN-γ and TNF-α mRNA in the spleen, kidney, liver, or lung.

### Comparison of pathology in MHC I^-/-^ and WT mice infected with *O*. *tsutsugamushi*

**Fig 7:**
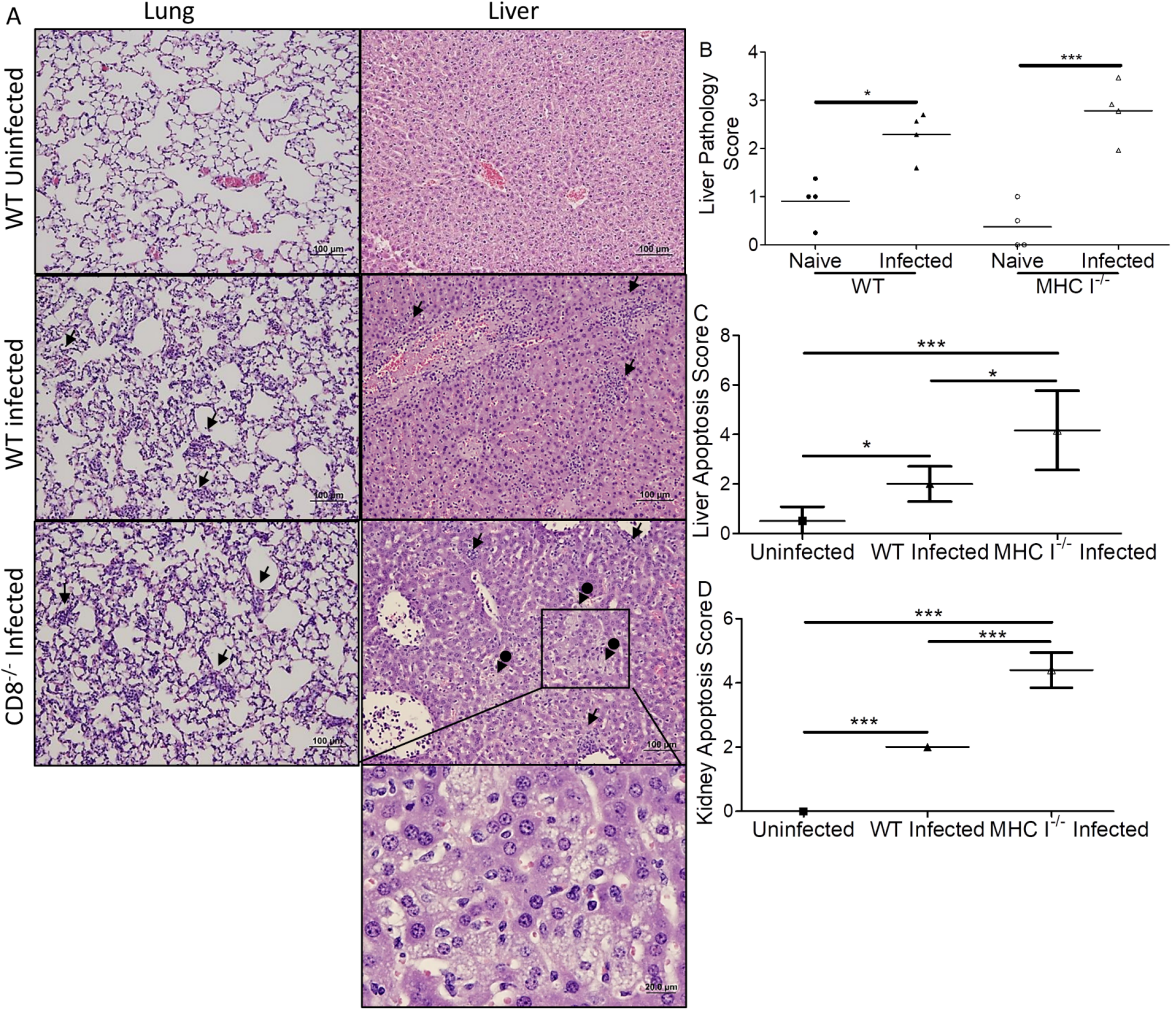
Histopathological comparison of MHC I^-/-^ mice and WT mice infected with *O*. *tsutsugamushi*. Foci of inflammation (arrows), including infiltration of macrophages and lymphocytes, were observed in infected mice (**A**, mag: 100x, 400x inset). Many apoptotic cells, possibly neutrophils, were observed in the liver of MHC I^-/-^ mice. Increased necrosis and steatosis (arrows with circle end) were observed in the livers of MHC I^-/-^ mice. Higher pathology scores indicating greater injury were observed in the livers of infected mice (**B**). There were significantly more apoptotic cells in the liver (**C**) and kidney (**D**) of MHC I^-/-^ mice than their WT counterparts. All infected mice had increased apoptosis compared to uninfected mice. *, p<0.05; ***, p<0.001, n = 8; each tissue sample was blindly scored by four experienced investigators.

A greater number of foci of cellular infiltrations were observed in the lung, liver, kidney and heart of WT mice than in matched MHC I^-/-^ mice (**Fig 7A, and S8 Fig**). The same scoring system as used previously revealed that infected MHC I^-/-^ mice had higher, but not significantly different, liver pathology scores than WT mice (2.78 vs 2.29, p = 0.10, **Fig 7B**). Both groups of *Orientia*-infected mice had significantly higher pathology scores than their uninfected counterparts. Serum analysis corroborated this finding with a trend in increased AST and ALT (**S4A & S4B Fig**). MHC I^-/-^ mice also had significantly lower ALB levels than CD8^-/-^ and WT mice (**S4C Fig**). In contrast with CD8^-/-^ mice, infected MHC I^-/-^ mice had significantly greater apoptotic scores than infected WT mice in both liver and kidney (p = 0.02, **Fig 7C & 7D and S5 Fig**). There was no significant difference between mRNA levels of *Bcl*-2 in the liver of infected WT and MHC I^-/-^ mice compared to corresponding uninfected mice at 11 dpi. Infected MHC I^-/-^ mice had lower but not statistically significant *Bcl*-2 mRNA levels than infected WT mice (**S8 Fig**).

### Adoptive transfer of immune CD8^+^ T lymphocytes or CD8 T cell-depleted splenocytes protected mice against *Orientia* infection

**Fig 8:**
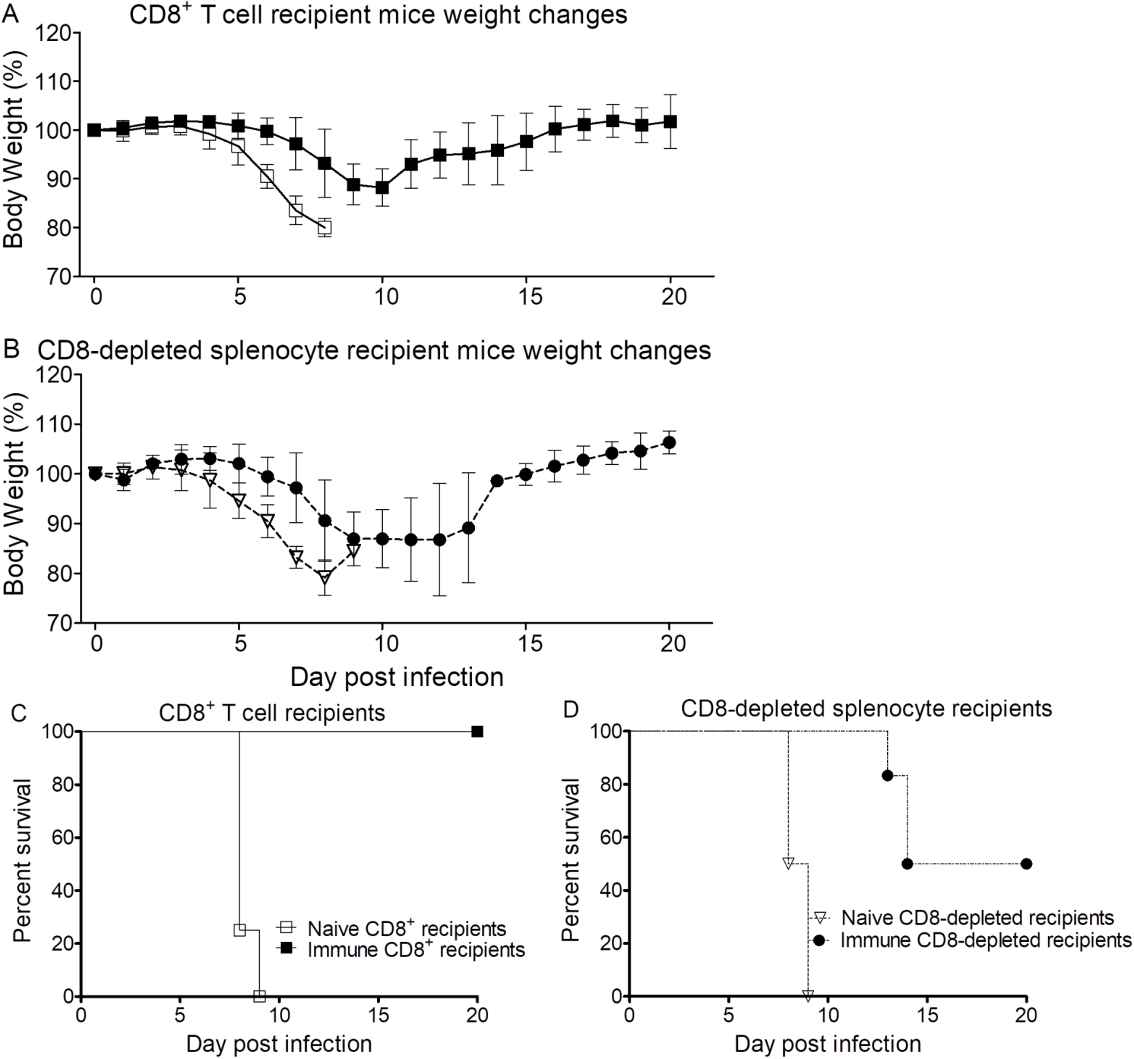
Body weight change and survival of WT mice adoptively transferred i.v. with different populations of splenocytes and challenged with *O*. *tsutsugamushi*. Naive CD8^+^ T cell recipient mice (open squares) began losing weight at 4 dpi with onset of other signs of illness at 6 dpi (**A**), and all were moribund between 8 and 9 dpi (**C**). Immune CD8^+^ T cell-recipient mice (solid squares) had delayed onset of illness that was milder, and they lost less weight between 7 and 10 dpi (**A)**, and all survived (**C**). We observed that naive CD8-depleted splenocyte recipient mice (open triangles) shared a similar course as the naive CD8^+^ T cell recipient group (**B** & **D**). Half of immune CD8-depleted splenocyte-recipient mice (solid circles) were moribund between 13 dpi and 14 dpi (**B** & **D**). Inoculation dose: 10 LD_50_ (8.25×10^7^ FFU), n = 5.

To further evaluate the roles of CD8^+^ T lymphocytes and other immune cells in scrub typhus, immune or naive CD8^+^ T cells or CD8 T cell-depleted splenocytes were adoptively transferred to WT C57BL/6J mice. We challenged the recipient mice with a 10 LD_50_ dose (8.25×10^7^ FFU) of *O*. *tsutsugamushi* Karp strain. All mice that received naive CD8^+^ T cells or naive CD8 T cell-depleted splenocytes were moribund between 8 and 9 dpi (**Fig 8**). Half of the mice that received immune CD8^+^ T cell-depleted splenocytes were moribund between 13 and 14 dpi. The onset of signs of illness was delayed when compared to mice that received naive CD8 T cell-depleted splenocytes. All mice that received immune CD8^+^ T cells survived. Compared to the other groups, these mice began showing signs of illness later, and their illness was much less severe. These mice lost ~12% or less of body weight, while the recipients of immune CD8 T cell-depleted splenocytes lost ~13% of body weight during infection after challenge with 10 LD_50_ of *O*. *tsutsugamushi*. Mice that received nonimmune CD8^+^ T cells or CD8 T cell-depleted splenocytes lost ~20% of their body weight. Immune CD8^+^ T cells were highly protective, and CD8 T cell-depleted splenocytes provided less, but significant, protection against the lethal dose challenge of *O*. *tsutsugamushi* with delayed onset and 50% survival. Even though CD8^+^ T cells are critical and provide effective protection, some other non-CD8^+^ immune cells also contribute to protection in *O*. *tsutsugamushi* infection.

## Discussion

There is still a large gap in understanding how *O*. *tsutsugamushi* invades, disseminates, and interacts within the host [12]. We discovered previously that host immunity is skewed towards T_h_ 1 responses from 12 dpi until 3 months post-infection [15]. Our flow cytometry data determined that more CD8^+^ T cells than CD4^+^ T cells were present in the spleen of infected mice after 12 dpi. We also found that CD4^+^CD25^+^Foxp3^+^ T_reg_ cells and levels of IL-10-producing T cells increased significantly from 6 dpi, which paralleled the change in body weight and increased bacterial loads. These studies contribute to the foundation of understanding the dynamic changes of host immunity during *O*. *tsutsugamushi* infection.

In our studies, IFN-γ and granzyme B mRNA levels in the liver of *O*. *tsutsugamushi*-infected CD8^-/-^ mice and MHC I^-/-^ mice were significantly higher than in infected WT mice, though both groups of KO mice had higher bacterial loads and mortality rates upon sublethal challenge (100% vs 0%) than their WT counterparts. IFN-γ is produced by multiple cell types including natural killer cells, natural killer T cells, CD4^+^ T cells, and CD8 T^+^ cells [46]. Both CD8^+^ T lymphocytes and NK cells produce granzyme B to mediate apoptosis in target cells [43]. We also observed higher mRNA levels of CXCL-10 in the liver of infected MHC I^-/-^ mice than in infected WT mice or uninfected MHC I^-/-^ mice. We did not observe the spike of CXCL-10 levels in the liver of CD8^-/-^ mice (S2 Fig). Secreted by several cell types including endothelial cells, CXCL-10 is an important chemokine for T cell accumulation under the influence of IFN-γ [47,48]. The increase of CXCL-10 mRNA levels in the liver of infected mice could be the reponse of infected endothelial cells to *O*. *tsutsugamushi*. Higher levels of IFN-γ in MHC I^-/-^ mice could induce higher levels of CXCL-10 than in WT mice. Although both groups of KO mice have a deficient cytotoxic CD8^+^ T cell response, other immune response cells are present and may have compensatory changes in their functional levels. We hypothesize that other non-CD8^+^ immune cells compensated by increased secretion of pro-inflammatory cytokines and granzyme although possibly not in the required location or functional conditions. Our studies demonstrated that even though there were enhanced levels of IFN-γ and granzyme B from other immune response cells, the host immune system nevertheless failed to control and eliminate the bacteria. Our RT-PCR results demonstrated no significant differences in the hepatic levels of TNF-α, IL-10, MCP-1, and MIP-2 between infected WT and CD8^-/-^ or MHC I^-/-^ mice.

Histopathological results demonstrated greater immune-inflammatory cell infiltration in different tissues of WT mice than in CD8^-/-^ mice and MHC I^-/-^ mice, but increased hepatic multifocal lesions and necrosis in CD8^-/-^ mice and MHC I^-/-^ mice. We postulate that the increased cellular infiltration was part of the host immune response to control the intracellular bacterial infection; however, this immune response, including upregulated IFN-γ and granzyme B, remained ineffective in CD8^-/-^ and MHC I^-/-^ mice. Our results suggested that the host immune responses contribute more to protective roles than causing pathologic effects. These findings were consistent with previous studies on *O*. *tsutsugamushi* and other rickettsiae [39,40,49]. We further examined the levels of alanine transaminase (ALT), aspartate transaminase (AST), and albumin (ALB) in serum of infected and uninfected WT, CD8^-/-^, and MHC I^-/-^ mice (S4 Fig.). Our blood chemistry results further confirmed that there was greater hepatocytic injury in both groups of knockout mice than WT mice. This study suggests the host mortality upon lethal-dose challenge was to a greater extent resultant of *O*. *tsutsugamushi* pathogenicity rather than host immunopathologic effects. Both higher apoptotic staining score and downregulation of the anti-apoptotic gene *Bcl*-2 mRNA levels confirmed that *Orientia* infection induced apoptosis in the host regardless of whether mice were WT or deficient in CD8 or MHC I [50]. *Bcl*-2 and other members in its family are critical for controlling the mitochondrial pathway of apoptosis after being activated by p53 tumor suppressor protein [51]. It could be a host defense mechanism that the balance of apoptosis was skewed towards pro-apoptotic pathways instead of anti-apoptotic pathways after *O*. *tsutsugamushi* infection. We further demonstrated that there was more apoptosis in the MHC I^-/-^ mice than WT mice and CD8^-/-^ mice. Our data support the hypothesis that NK cells in MHC I^-/-^ mice may induce more apoptosis in the tissue after *Orientia* infection because MHC-I also functions as an inhibitor of NK cells’ cytotoxicity *in vivo* [52,53]. More studies are necessary to understand the mechanisms behind this.

Our adoptive transfer experiment provided direct evidence of the contribution of CD8^+^ T lymphocytes and the mixture of the non-CD8 immune cells including potentially CD4^+^ T cells, macrophages, NK cells, NKT cells, and neutrophils in immunity to scrub typhus. Our studies demonstrated that in addition to immune CD8^+^ T lymphocytes other non-CD8 immune cells also provide protection against scrub typhus, even though the protection by immune CD8^+^ T cells was more effective than the mixture of non-CD8 cells. Neither naive CD8^+^ T cells nor naive CD8^+^ cell-depleted splenocytes provided any protection. Our results utilizing the i.v. route of infection supports the study of Hauptmann et al (with the i.p. and footpad *Orientia* challenge routes and i.p. adoptive transfer)[17], which found a critical role for CD8^+^ T cells, but it also implicates the contribution of other non-CD8^+^ immune cells in protection against *O*. *tsutsugamushi*. Further studies are necessary to understand the roles of those non-CD8^+^ immune cells in host immunity against *O*. *tsutsugamushi*. Previously, Kodama et al. reported the protective function of immune CD4^+^ T cells to i.p. inoculation of *O*. *tsutsugamushi* infection [34]. Given that i.p. and s.c. footpad inoculations do not mimic the human infection as the i.v. and i.d. models do [24,25], validating the role of immune cells by using a well-developed mouse model and high-purity CD8^+^ T cells (91% purity) in this study is important for our understanding of host immunity to *Orientia* infection.

The utilization of inaccurate disease models of scrub typhus fundamentally impedes the necessary studies to understand host-pathogen interactions. *Orientia tsutsugamushi* causes disseminated endothelial infection and multifocal vasculitis in lung and brain of human patients. Patients suffer interstitial pneumonitis, hepatic damage, encephalitis, and disseminated lymphohistiocytic vasculitis during scrub typhus [18,22,54]. However, the historic animal infection with *O*. *tsutsugamushi*, first employed more than 50 years ago, involves i.p. injection of *O*. *tsutsugamushi* into mice, which substantially limits the pathogens and lesions to the peritoneal cavity [23,55]. Continuous proliferation of *O*. *tsutsugamushi* in mesothelial cells and peritoneal macrophages, enlargement of the spleen, hepatic lesions, peritonitis, and limited dissemination occur in these i.p. infected mice [23,24,56,57,58]. This widely used i.p. route produces an infection of the peritoneal cavity that results in fatal *Orientia* peritonitis not observed in scrub typhus infection in human patients [18]. The report of Hauptmann used this less appropriate mouse model in their CD8^+^ T cell adoptive transfer studies. Natural chigger-bite transmission results in intradermal, not subcutaneous inoculation, as a portion of footpad inoculation produces. Recently, our laboratory has developed new i.v. and i.d. inoculation mouse models for *Orientia* infection, which better mimic the pathogen distribution, pathology and immunology of human scrub typhus patients, namely accurately reflecting the i.d. site of chigger feeding inoculation of *O*. *tsutsugamushi* and hematogenous dissemination, respectively. Our data regarding the histopathology and cytokines in relevant organs such as lung and liver confirmed that the i.v. and i.d. inoculated mouse models developed in our laboratory are more appropriate for the study representing systemic disseminated and mite-inoculated scrub typhus, respectively [15,24,25].

In summary, we have demonstrated that CD8^+^ T cells play a critical protective role in the host immune response against *O*. *tsutsugamushi* infection in our hematogenously disseminated endothelial cell and macrophage target murine model of scrub typhus. However, we also determined that other immune response cells in addition to CD8^+^ T cells contribute protective effects during scrub typhus. Our animal models and experimental design more appropriately mimicked the development of disease and host immunity; therefore, these results are an important contribution to the understanding of the host immune responses during *O*. *tsutsugamushi* infection. Our studies proved that CD8^+^ T cells together with other immune response cells protect the host from *O*. *tsutsugamushi* infection by eliminating intracellular organisms from endothelial cells and macrophages. We also further demonstrated that pro-inflammatory cytokines, such as IFN-γ, and granzyme B cannot control and clear the intracellular bacteria in the absence of CD8^+^ T cells or MHC I. Although there were significantly greater cellular infiltrations in infected WT mice, our histopathologic analysis determined that CD8^-/-^ mice had a higher hepatic pathology score, indicating greater tissue damage than in WT mice and MHC I^-/-^ mice. Further studies, including survival of mice deficient in CD4^+^ T cells and/or other immune cells, during scrub typhus will determine the protective mechanisms in greater detail. The understanding of these mechanisms will facilitate development of innovative management for *O*. *tsutsugamushi* infections by targeting stimulation of immunity in CD8^+^ T cells and other host immune components.

## Supporting information

S1 Table Primers of murine genes for qRT-PCR. (DOCX)

S1 Fig Levels of T_reg_ cells and IL-10 producing T cells in *Orientia-*infected mice. From day 6 after *Orientia* infection, more CD4^+^CD25^+^FoxP3^+^ T_reg_ cells (**A**) and IL-10 producing CD3^+^ T cells (**B**) were detected in both sublethal and lethal dose challenged mice than uninfected control mice. The levels of both T_reg_ cells and IL-10 producing CD3^+^ T cells peaked on day 6. Data are expressed as mean ± SD. *, p<0.05; ***, p<0.001.
(TIF)

S2 Fig CXCL-10 and TNF-α mRNA levels in the liver of CD8^-/-^ and WT mice infected with *O*. *tsutsugamushi*. Infected WT mice had greater mRNA levels of CXCL-10 than infected CD8^-/-^ mice and uninfected controls (**A**). No statistically significant difference was detected between levels of TNF-α among CD8^-/-^ and WT mice (**B**). Data are shown as mean ± SD in each group and presented as relative mRNA levels with the 2^-Δ ΔCt^ of housekeeping genes normalization method. **, p<0.01.
(TIF)

S3 Fig IL-10, MCP-1 and MIP-2 mRNA levels in the liver of CD8^-/-^, MHC I^-/-^ and WT mice infected with *O*. *tsutsugamushi*. No statistical significance was detected in the mRNA levels of IL-10 (**A**), MCP-1 (**B**) and MIP-2 (**C**) among CD8^-/-^, MHC I^-/-^ and WT mice. Data are shown as mean ± SD in each group and presented as relative mRNA levels normalized to housekeeping genes using 2^-Δ ΔCt^ method.
(TIF)

S4 Fig Serum enzymes and albumin levels in CD8^-/-^, MHC I^-/-^ and WT mice infected with *O*. *tsutsugamushi*. We observed significantly greater ALT and AST levels in the sera of infected mice than uninfected control (**A** and **B**). Both infected CD8^-/-^ and MHC I^-/-^ mice had greater, but not statistically different, levels than WT mice. Infected mice had lower albumin levels than uninfected control. MHC I^-/-^ mice had significantly lower albumin levels than CD8^-/-^ and WT mice (**C**). *, p<0.05; **, p<0.01, ***, p<0.001; n = 3.
(TIF)

S5 Fig Apoptosis in the kidney and liver of CD8^-/-^, MHC I^-/-^ and WT mice infected with *O*. *tsutsugamushi*. There were significantly lower mRNA levels of *Bcl*-2 in the liver of infected WT and CD8^-/-^ mice than in the corresponding uninfected mice (**A**). No difference was detected between infected WT and CD8^-/-^ mice at 12 dpi (**A**). Representative immunohistochemical staining demonstrated more apoptosis in infected mice, especially CD8^-/-^ and MHC I^-/-^ mice (**B**). Data are shown as mean ± SD in each group and presented as relative mRNA levels normalized to housekeeping gene with the 2^-Δ ΔCt^ method. *, p<0.05; **, p<0.01.
(TIF)

S6 Fig Body weight change of MHC I^-/-^ mice and WT mice infected with *O*. *tsutsugamushi*. Infected mice began losing weight at 9 dpi coincident with signs of illness. Both WT (solid circles) and MHC I^-/-^ mice (open circles) shared the same weight loss trend until 11 dpi, when WT infected mice, but not MHC I^-/-^ infected mice, began to recover and gain weight.
(TIF)

S7 Fig CXCL-10 and TNF-α mRNAlevel in the liver of MHC I^-/-^ mice and WT mice infected with *O*. *tsutsugamushi*. There were significantly higher mRNA levels of CXCL-10 in the liver of infected MHC I^-/-^ mice than in the WT mice at 11 dpi. (**A**). No statistical significance was detected in the levels of TNF-α between MHC I^-/-^ and WT mice but MHC I^-/-^ mice had increased TNF-α mRNA levels than uninfected control (**B**). Data are shown as mean ± SD in each group and presented as relative mRNA levels with the 2^-Δ ΔCt^ of housekeeping genes normalization method. *, p<0.05; **, p<0.01.
(TIF)

S8 Fig Histopathological comparison of MHC I^-/-^ and WT mice infected with *O*. *tsutsugamushi*. Fewer foci of inflammation (arrows), including infiltration of macrophages and lymphocytes, were observed in MHC I^-/-^ mice (**A**). There was no significant difference between mRNA levels of *Bcl*-2 in the liver of infected WT and MHC I^-/-^ mice compared to corresponding uninfected mice at 11 dpi (**B**). Infected MHC I^-/-^ mice had lower but not significantly different *Bcl*-2 mRNA levels than infected WT mice (B, p = 0.067).
(TIF)
