# Correction: CD8^+^ T cells provide immune protection against murine disseminated endotheliotropic *Orientia tsutsugamushi* infection

**DOI:** 10.1371/journal.pntd.0006127

**Published:** 2017-12-06

**Authors:** Guang Xu, Nicole L. Mendell, Yuejin Liang, Thomas R. Shelite, Yenny Goez-Rivillas, Lynn Soong, Donald H. Bouyer, David H. Walker

Fig 7A is incorrect. The label “CD8^-/-^ Infected” should read “MHC I^-/-^ Infected”. The authors have provided a corrected version here.

**Fig 7:**
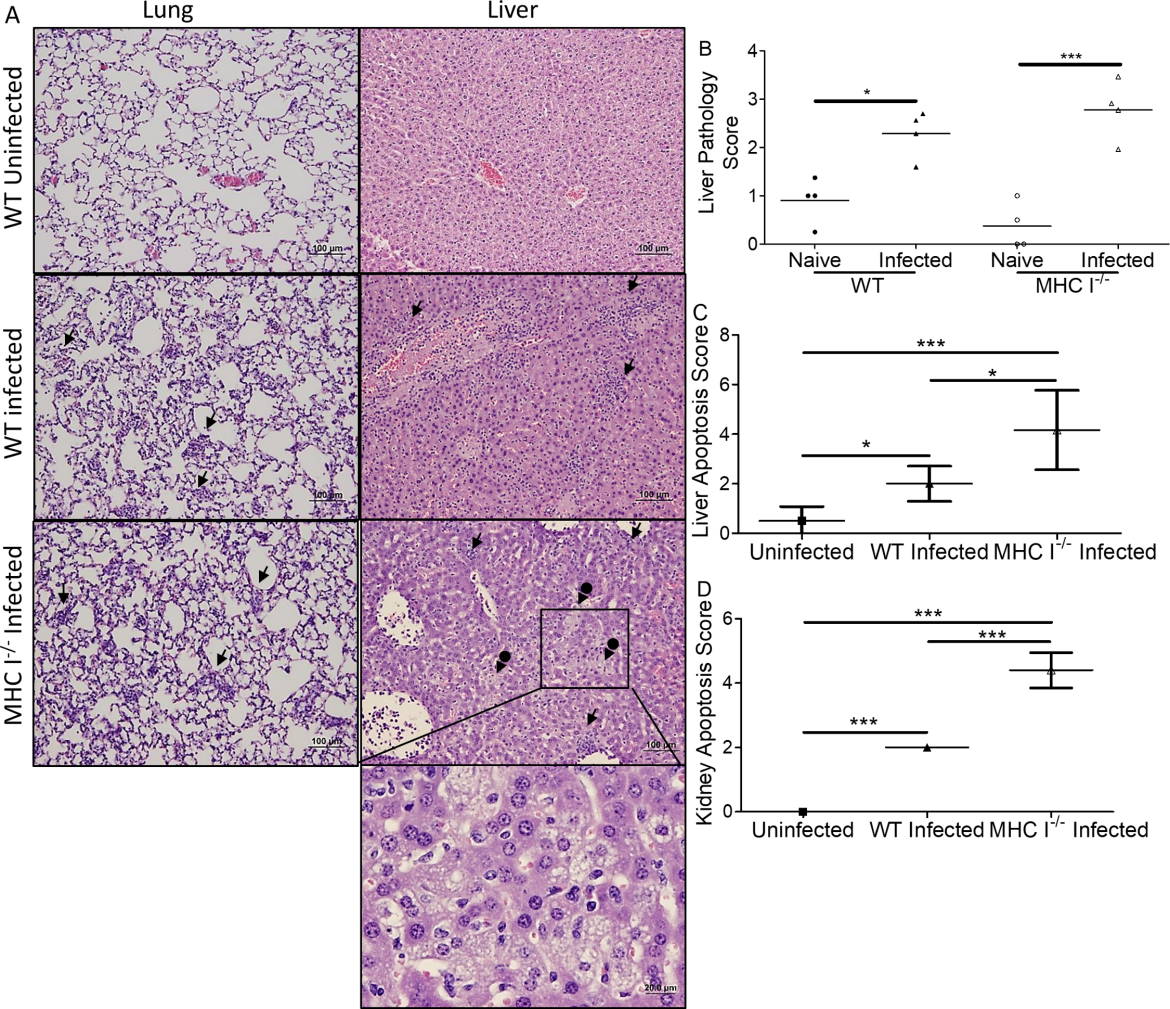
Histopathological comparison of MHC I^-/-^ mice and WT mice infected with *O. tsutsugamushi*. Foci of inflammation (arrows), including infiltration of macrophages and lymphocytes, were observed in infected mice (**A**, mag: 100x, 400x inset). Many apoptotic cells, possibly neutrophils, were observed in the liver of MHC I^-/-^ mice. Increased necrosis and steatosis (arrows with circle end) were observed in the livers of MHC I^-/-^ mice. Higher pathology scores indicating greater injury were observed in the livers of infected mice (**B**). There were significantly more apoptotic cells in the liver (**C**) and kidney (**D**) of MHC I^-/-^ mice than their WT counterparts. All infected mice had increased apoptosis compared to uninfected mice. *, p<0.05; ***, p<0.001, n = 8; each tissue sample was blindly scored by four experienced investigators.
