# Supplementary material for: CD8+ T cells provide immune protection against murine disseminated endotheliotropic Orientia tsutsugamushi infection
Source: PLoS Negl Trop Dis. 2017 Jul 19;11(7):e0005763. doi: 10.1371/journal.pntd.0005763 (PMC5536391; doi:10.1371/journal.pntd.0005763)
Supplement: S1 Table — (DOCX) [file pntd.0005763.s001.docx]

| **S1 Table. Primers of murine genes for qRT-PCR** | | |
| --- | --- | --- |
| GAPDH | Forward 5’-TGGAAAGCTGTGGCGTGAT-3’ | Reverse 5’-TGCTTCACCACCTTCTTGAT-3’ |
| IFN-γ | Forward 5’-ATGAACGCTACACACTGCATC-3’ | Reverse 5’-CCATCCTTTTGCCAGTTCCTC-3’ |
| TNF-α | Forward 5’-ATAGCTCCCAGAAAAGCAAGC-3’ | Reverse 5’-TTGGTCCTTAGCCACTCCTTC-3’ |
| CXCL-10 | Forward 5’-CCAAGTGCTGCCGTCATTTTC-3’ | Reverse 5’-GGCTCGCAGGGATGATTTCAA-3’ |
| *Bcl*-2 | Forward 5’-ATGCCTTTGTGGAACTATATGGC-3’ | Reverse 5’-GGTATGCACCCAGAGTGATGC-3’ |
| IL-10 | Forward 5’-GCTCTTACTGACTGGCATGAG-3’ | Reverse 5’- CGCAGCTCTAGGAGCATGTG-3’ |
| Granzyme B | Forward 5’-CCACTCTCGACCCTACATGG-3’ | Reverse 5’-GGCCCCCAAAGTGACATTTATT-3’ |
| MCP-1 | Forward 5’-TTAAAAACCTGGATCGGAACCAA-3’ | Reverse 5’-GCATTAGCTTCAGATTTACGGGT-3’ |
| MIP-2 | Forward 5’-CCAACCACCAGGCTACAGG-3’ | Reverse 5’-GCGTCACACTCAAGCTCTG-3’ |
|  |  |  |
