# Supplementary figures and images for: CD8+ T cells provide immune protection against murine disseminated endotheliotropic Orientia tsutsugamushi infection
Source: PLoS Negl Trop Dis. 2017 Jul 19;11(7):e0005763. doi: 10.1371/journal.pntd.0005763 (PMC5536391; doi:10.1371/journal.pntd.0005763)

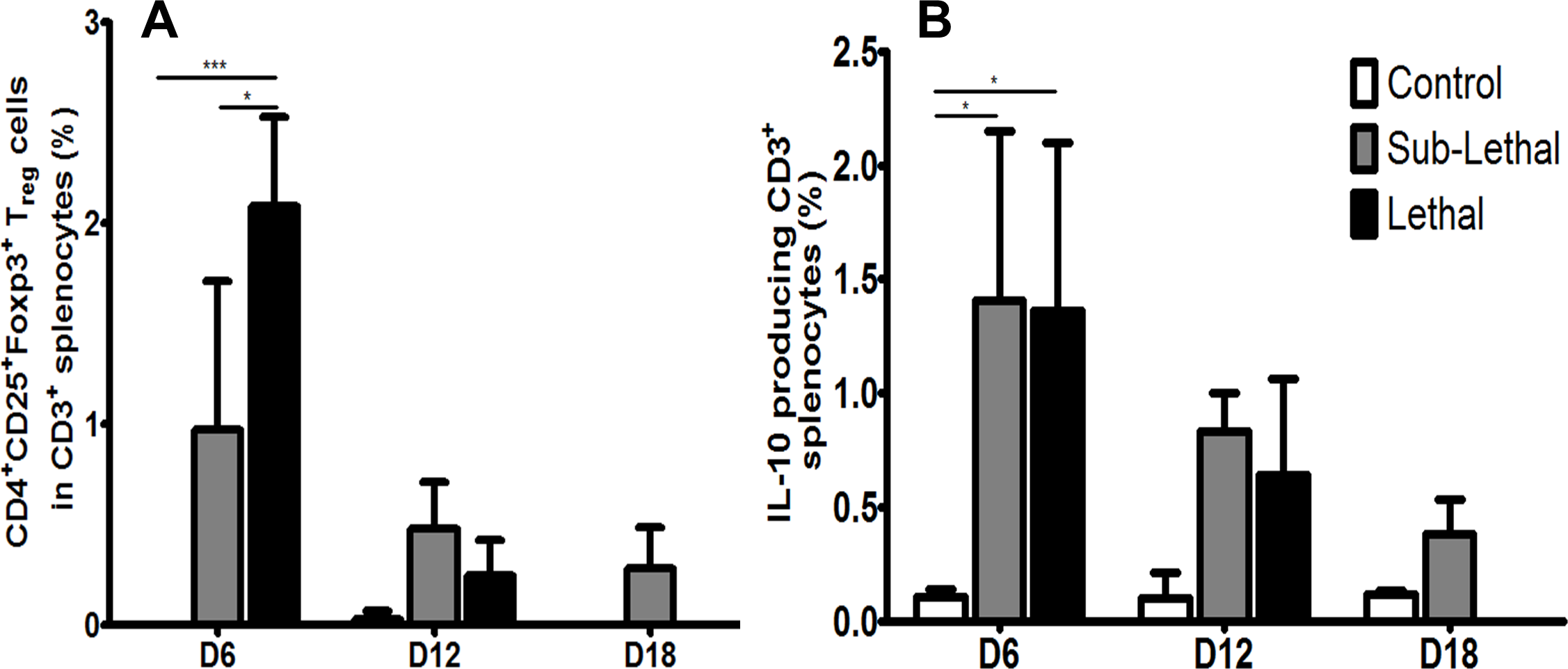

Supplement: S1 Fig — From day 6 after Orientia infection, more CD4+CD25+FoxP3+ Treg cells (A) and IL-10 producing CD3+ T cells (B) were detected in both sublethal and lethal dose challenged mice than uninfected control mice. The levels of both Treg cells and IL-10 producing CD3+ T cells peaked on day 6. Data are expressed as mean ± SD. *, p<0.05; ***, p<0.001. (TIF) [file pntd.0005763.s002.tif]

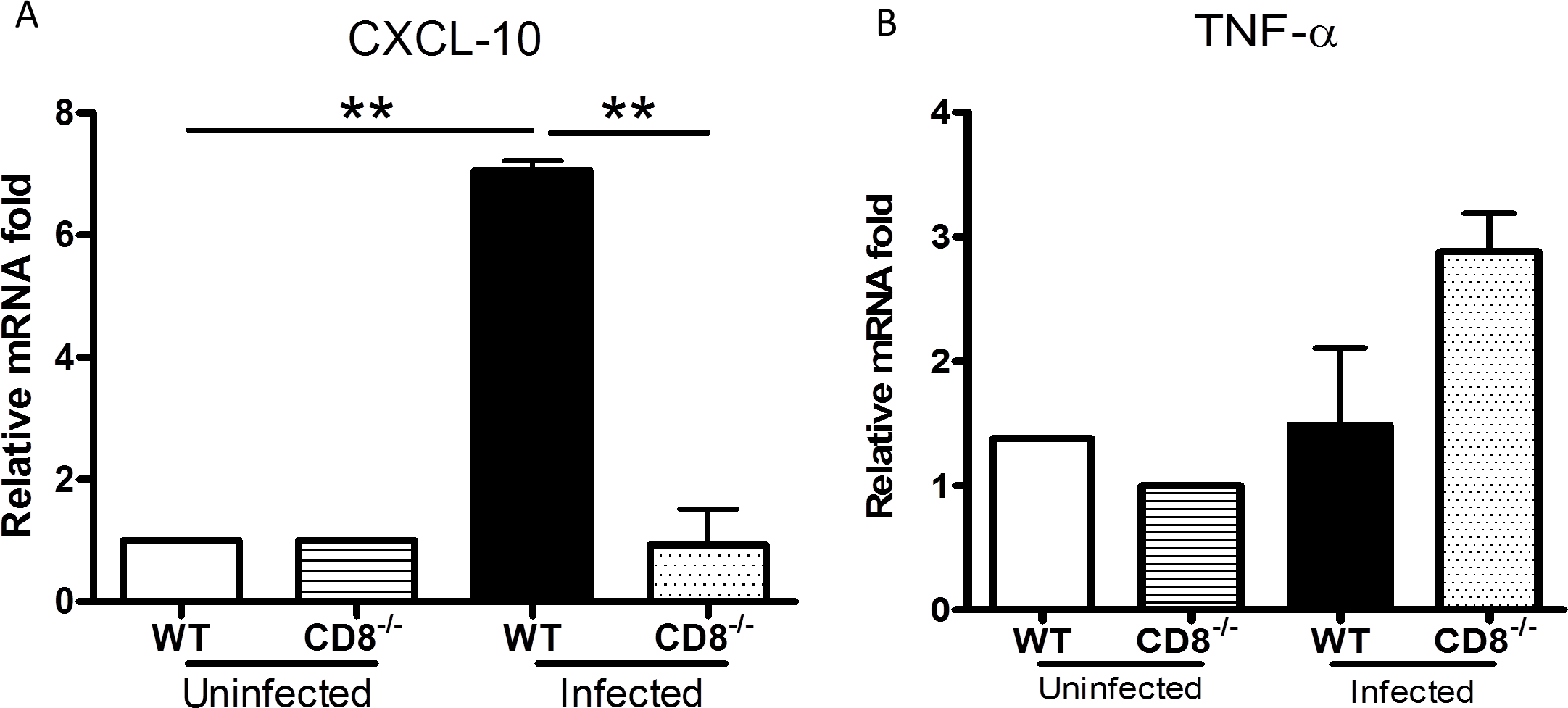

Supplement: S2 Fig — Infected WT mice had greater mRNA levels of CXCL-10 than infected CD8-/- mice and uninfected controls (A). No statistically significant difference was detected between levels of TNF-α among CD8-/- and WT mice (B). Data are shown as mean ± SD in each group and presented as relative mRNA levels with the 2-Δ ΔCt of housekeeping genes normalization method. **, p<0.01. (TIF) [file pntd.0005763.s003.tif]

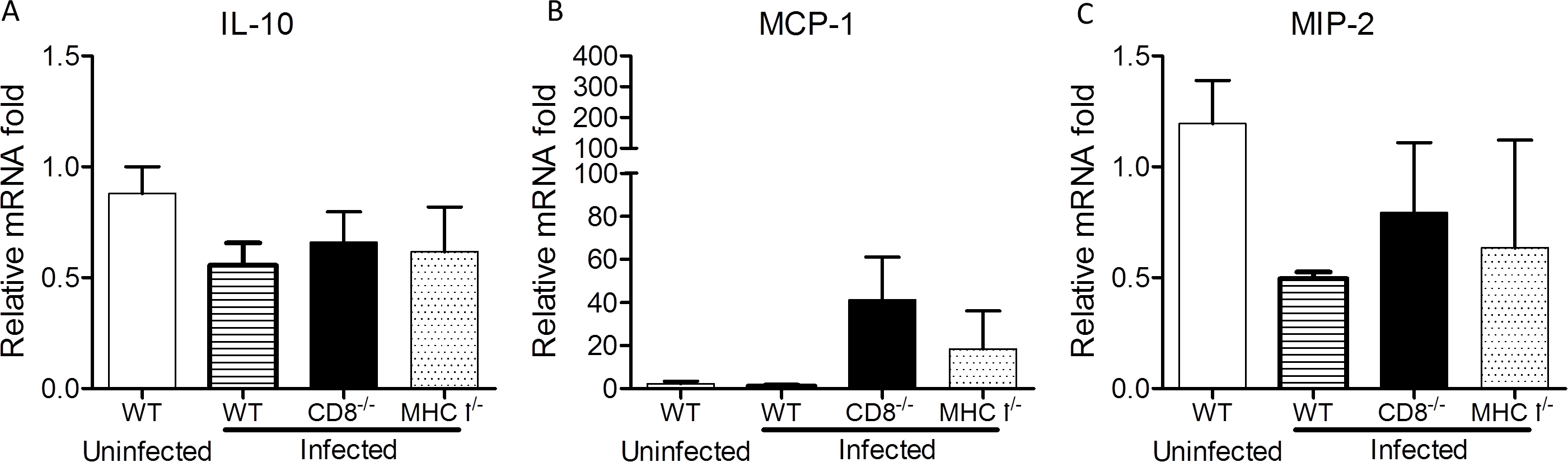

Supplement: S3 Fig — No statistical significance was detected in the mRNA levels of IL-10 (A), MCP-1 (B) and MIP-2 (C) among CD8-/-, MHC I-/- and WT mice. Data are shown as mean ± SD in each group and presented as relative mRNA levels normalized to housekeeping genes using 2-Δ ΔCt method. (TIF) [file pntd.0005763.s004.tif]

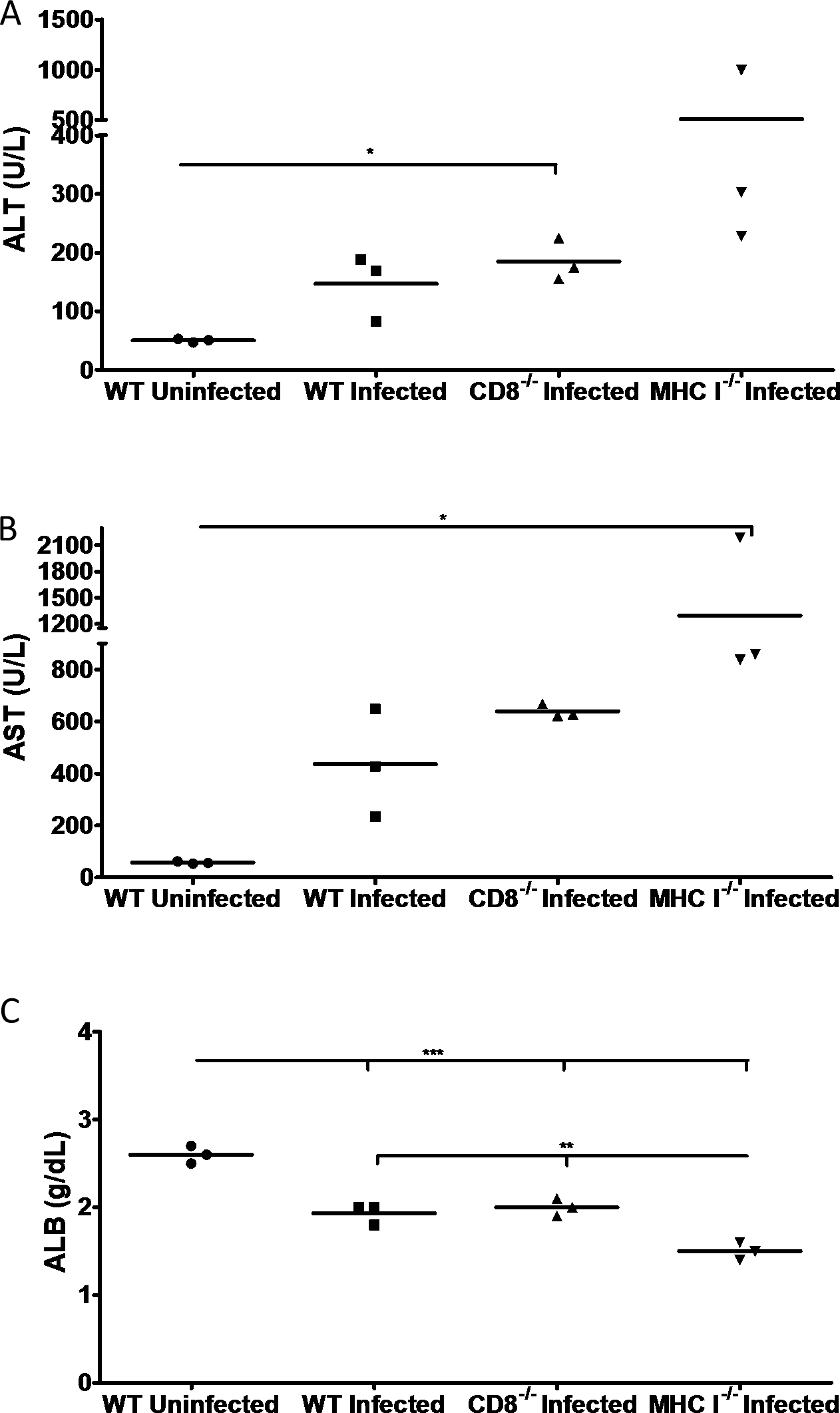

Supplement: S4 Fig — We observed significantly greater ALT and AST levels in the sera of infected mice than uninfected control (A and B). Both infected CD8-/- and MHC I-/- mice had greater, but not statistically different, levels than WT mice. Infected mice had lower albumin levels than uninfected control. MHC I-/- mice had significantly lower albumin levels than CD8-/- and WT mice (C). *, p<0.05; **, p<0.01, ***, p<0.001; n = 3. (TIF) [file pntd.0005763.s005.tif]

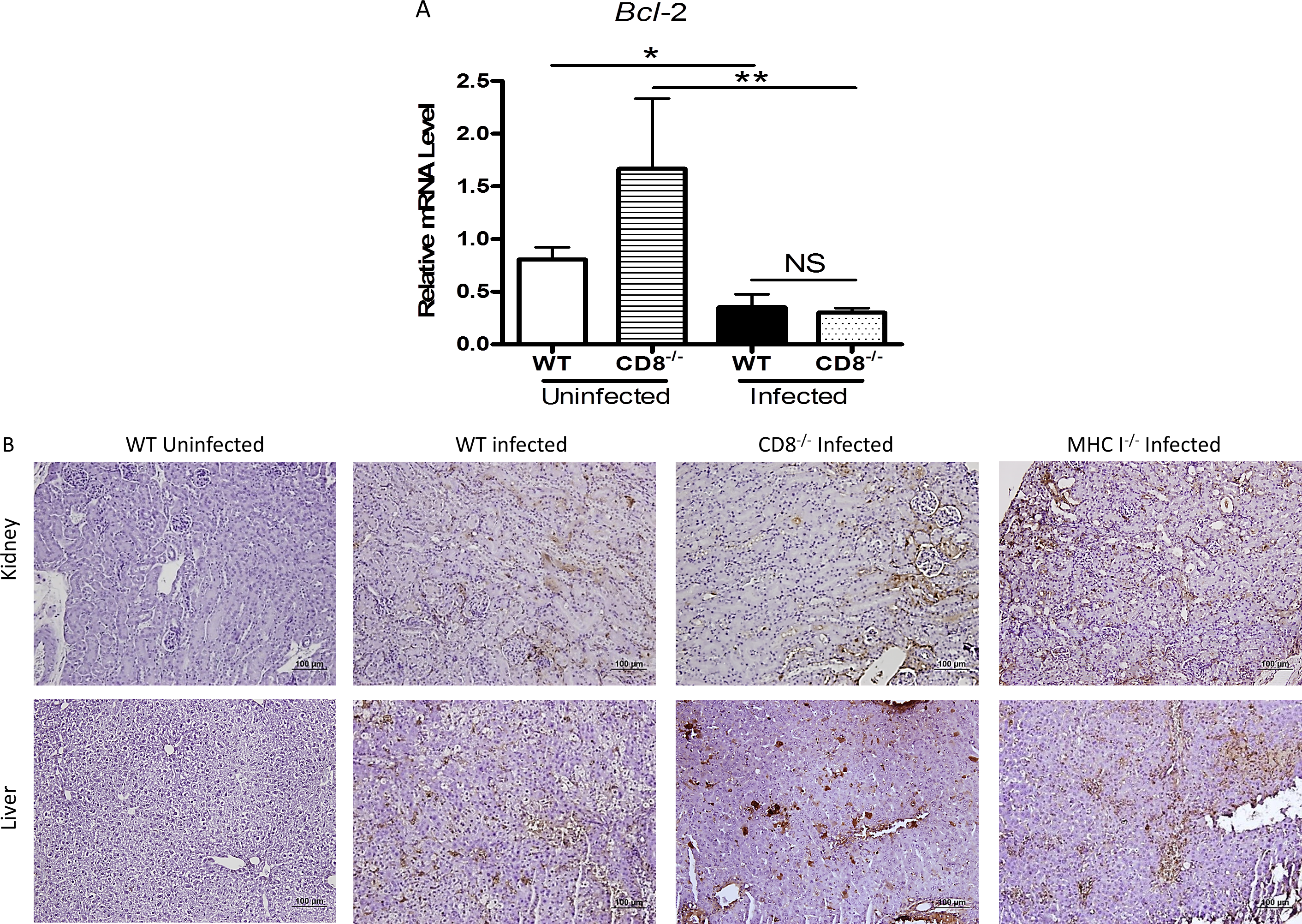

Supplement: S5 Fig — There were significantly lower mRNA levels of Bcl-2 in the liver of infected WT and CD8-/- mice than in the corresponding uninfected mice (A). No difference was detected between infected WT and CD8-/- mice at 12 dpi (A). Representative immunohistochemical staining demonstrated more apoptosis in infected mice, especially CD8-/- and MHC I-/- mice (B). Data are shown as mean ± SD in each group and presented as relative mRNA levels normalized to housekeeping gene with the 2-Δ ΔCt method. *, p<0.05; **, p<0.01. (TIF) [file pntd.0005763.s006.tif]

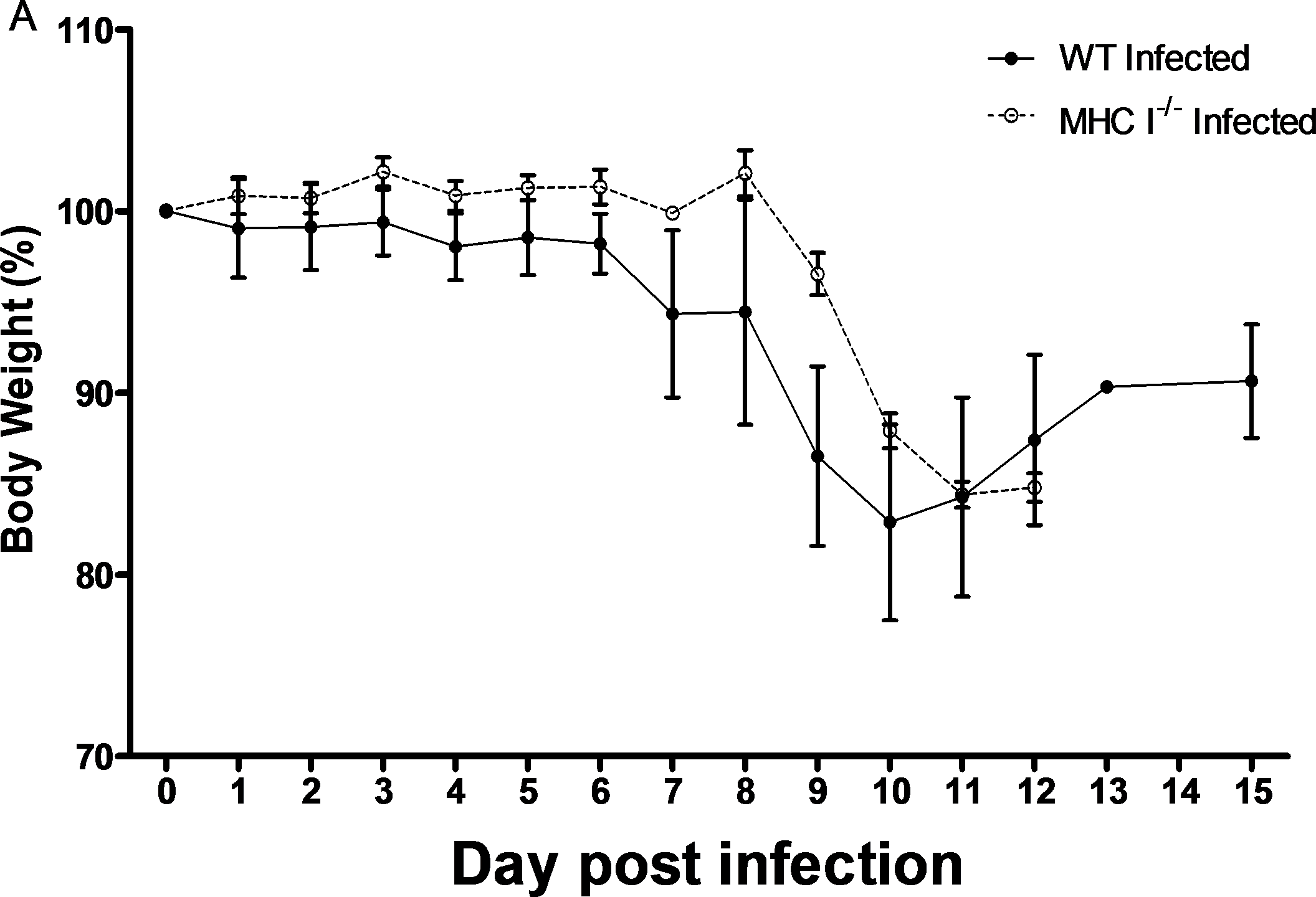

Supplement: S6 Fig — Infected mice began losing weight at 9 dpi coincident with signs of illness. Both WT (solid circles) and MHC I-/- mice (open circles) shared the same weight loss trend until 11 dpi, when WT infected mice, but not MHC I-/- infected mice, began to recover and gain weight. (TIF) [file pntd.0005763.s007.tif]

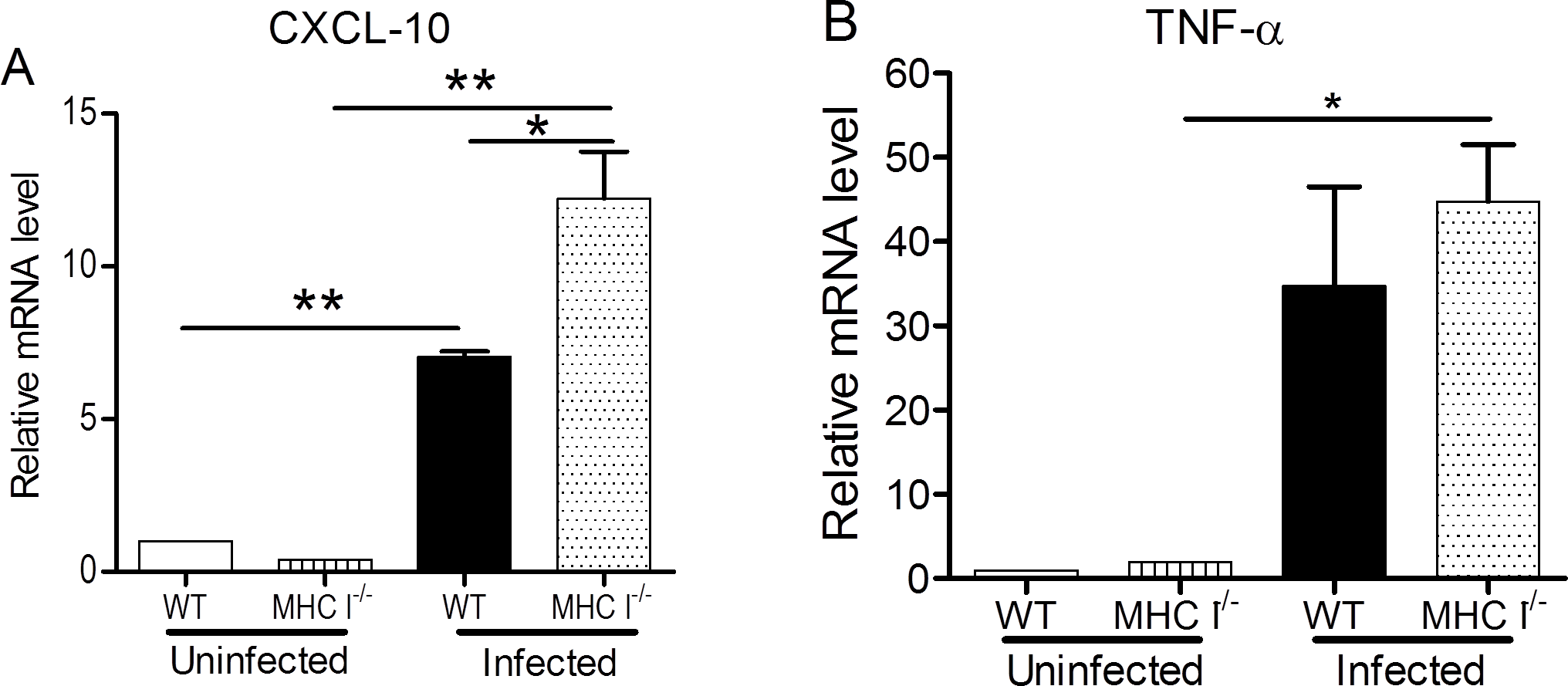

Supplement: S7 Fig — There were significantly higher mRNA levels of CXCL-10 in the liver of infected MHC I-/- mice than in the WT mice at 11 dpi. (A). No statistical significance was detected in the levels of TNF-α between MHC I-/- and WT mice but MHC I-/- mice had increased TNF-α mRNA levels than uninfected control (B). Data are shown as mean ± SD in each group and presented as relative mRNA levels with the 2-Δ ΔCt of housekeeping genes normalization method. *, p<0.05; **, p<0.01. (TIF) [file pntd.0005763.s008.tif]

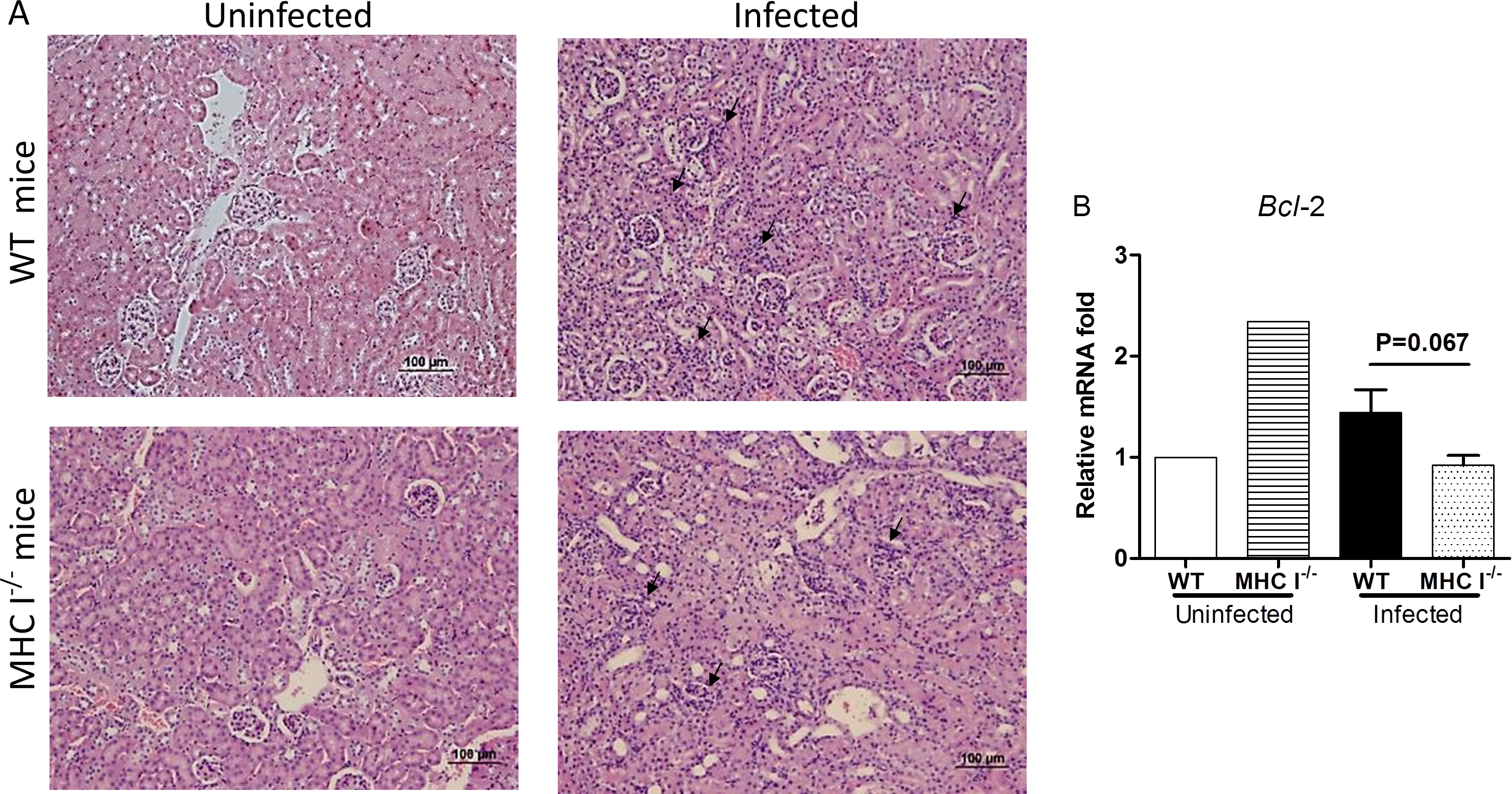

Supplement: S8 Fig — Fewer foci of inflammation (arrows), including infiltration of macrophages and lymphocytes, were observed in MHC I-/- mice (A). There was no significant difference between mRNA levels of Bcl-2 in the liver of infected WT and MHC I-/- mice compared to corresponding uninfected mice at 11 dpi (B). Infected MHC I-/- mice had lower but not significantly different Bcl-2 mRNA levels than infected WT mice (B, p = 0.067). (TIF) [file pntd.0005763.s009.tif]
